# Supplementary material for: Disparate estimates of intrinsic productivity for Antarctic krill across small spatial scales under a rapidly changing ocean
Source: PLoS One. 2026 Jan 30;21(1):e0322671. doi: 10.1371/journal.pone.0322671 (PMC12857959; doi:10.1371/journal.pone.0322671)
Supplement: S1 File — (PDF) [file pone.0322671.s001.pdf]

Supporting Information 1

Disparate estimates of intrinsic productivity for Antarctic krill (Euphausia superba) across small spatial scales under a rapidly changing ocean.

12 December, 2025

Contents

CONTEXT **2**

METHODOLOGY **2**
Monitoring Data (SISO Program) . . . 2
LBSPR Modeling . . . 2

RESULTS **8**
Model Perfomance . . . 8
Comparing producivity between years and Stratas . . . 11
Sensitivity and perfomance analysis . . . 14
Analysis to three Subareas (48.1, 48.2 and 48.3) . . . 21

GLOSSARY **26**

CODE REPOSITORY **28**

REFERENCES **28**

CONTEXT

This supplementary material with essential formulas, datasets, and code snippets is delivered for the application of the LBSPR model in studying krill populations within the West Antarctic Peninsula (WAP) region. The LBSPR model, pivotal for unraveling krill dynamics, is reinforced by mathematical expressions elucidating key parameters and environmental influences. Curated datasets provide valuable insights into factors shaping krill habitats. With a strong emphasis on reproducibility, this resource ensures that findings can undergo independent validation, fostering trust in scientific outcomes. Transparent documentation of methods and assumptions promotes understanding and scrutiny of the analysis process by peers.

METHODOLOGY

Monitoring Data (SISO Program)

For this analysis, data from the monitoring of the krill fishery were used, which have been systematically collected on board fishing vessels by the CCAMLR SISO (Scheme of International Scientific Observation) program. Krill sizes compositions were obtained from the entire area 48.1, which was combined in each management stratum defined at 2.1 section (Figure 1). For this analysis, 1,266,267 krill individuals were measured from fishery activity, the majority (~75%) from the Bransfield, Elephant and SouthWest strata.

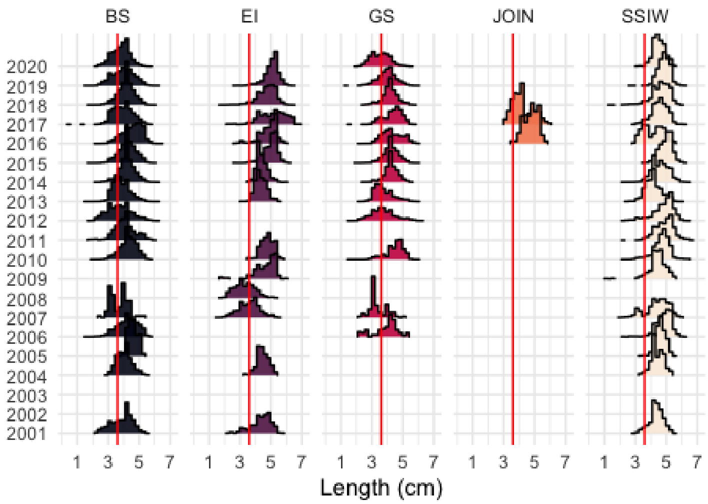

Figure 1: Sizes compositions from SISO program monitoring krill fishery by strata (BS=Brainsfield Strait, EI= Elephant Island, GS= Gerlache Strait, JOIN= Joinville Island, SSWI= South West). Red line represent recruit size

The information gaps (years without sizes composition data) are not calculated because there is no autocorrelation between years, but singular estimators over time.

LBSPR Modeling

Loading packages

```
## Installing the Package The LBSPR package is now
## available on CRAN: install.packages('LBSPR')
## install.packages('devtools')
## devtools::install_github('AdrianHordyk/LBSPR') load the
## package
```

```r
library(LBSPR)
library(devtools)  #to install_github
library(dplyr)
library(tidyr)
library(ggplot2)
library(stringr)
library(ggpubr)
library(kableExtra)
library(hrbrthemes)
library(ggthemes)
library(tidyverse)
```

Biological and fishery parameters krill

```r
# Create a new LB_pars Object To create a new LB_pars
# object you use the new function:
MyPars <- new("LB_pars")

# You can see the elements or slots of the LB_pars object
# using the slotNames function: slotNames(MyPars)
```

The model needs specifications related to both biological and fishery parameters according to species evaluated. In a descriptive way, the main parameter sets are described as follows;

Biology

- von Bertalanffy asymptotic length `Linf`
- M/K ratio (natural mortality)divided by von Bertalanffy K coefficient) `MK`
- Length at 50% maturity (`L50`)
- Length at 95% maturity (`L95`)

Fishery

- Length at 50% selectivity (`SL50`)
- Length at 95% selectivity (`SL95`)
- Biological Reference Point (BRP). F/M ratio (`FM`) or Spawning Potential Ratio (`SPR`). If you specify both, the F/M value will be ignored.

Size Classes

-Width of the length classes (`BinWidth`)

```r
# MyPars <- new('LB_pars') A blank LB_pars object created
# Default values have been set for some parameters
MyPars@Species <- "Euphausia superba"
MyPars@Linf <- 60
MyPars@L50 <- 34
MyPars@L95 <- 55  # verrificar bibliografia
MyPars@MK <- 0.4/0.45


# Explotation
MyPars@SL50 <- 40  #numeric() #1
MyPars@SL95 <- 56  #numeric() #27
MyPars@SPR <- 0.75  #numeric()# ###cambia el numero 0.4 a en blanco
MyPars@BinWidth <- 1
# MyPars@FM <- 1
```

```r
MyPars@Walpha <- 1
MyPars@Wbeta <- 3.0637  #r2 = 0.9651

MyPars@BinWidth <- 1
MyPars@BinMax <- 70
MyPars@BinMin <- 0
MyPars@L_units <- "mm"
```

These parameters were taken from previous works about krill life history and fishery (Maschette et al., 2021; Thanassekos et al., 2014), which are described in Table 1.

Table 1: Krill biological and fishery parameters

Description	Value
VB asymptotic length	60
Maturity 50%	34
Maturity 95%	55
M/K Ratio	0.889
Selectivity 50%	40
Selectivity 95%	56
SPR	0.75
a (Length-Weight Relation)	1
b (Length-Weight Relation)	3.0637
Bin Min	0
Bin Max	70
Units	mm

Model Estimation LBSPR

Recent work has shown that under equilibrium conditions (that is, constant F and no recruitment variability) and assuming the von Bertalanffy growth equation, constant natural mortality for all ages, and logistic or jack-knife selectivity, standardization of the composition of lengths of two populations with the same ratio of natural mortality to growth rate (M/k) and the same ratio of mortality by fishing to natural mortality (F/M) will be identical (A. R. Hordyk et al., 2016). Extension of this model to incorporate length-at-age variability and logistic selectivity confirms that, at equilibrium, the composition of the predicted duration of catch of an exploited population is primarily determined by the ratios of M/k and F/M. The analytical models developed in A. Hordyk et al. (2014) suggest that with knowledge of the asymptotic von Bertalanffy length L_∞ and the coefficient of variation in CVL_∞, the ratio of total mortality to the von Bertalanffy growth coefficient (Z/k) for a given population can be estimated from a representative sample of the size structure of the catch. If M/k (or parameters) is also known, then the results of A. R. Hordyk et al. (2016) suggest that it is possible to estimate F/M from the composition of the catch. Often the F/M ratio has been used as a biological reference point when is 1 (Zhou et al., 2012).

The LBSPR model requires the following parameters: an estimate of the M/k ratio, L_∞, CVL_∞, and knowledge of maturity by length (maturity ogive), both set parameters know in krill. This model uses data on composition by catch sizes to estimate intrinsic productivity or Spawning Potential Ratio (SPR). This concept was extracted from Goodyear (1993), where the ratio of lifetime average egg production per recruit (EPR) was calculated for fished and no fished (virgin condition) resources. An algorithm route for the calculation of the SPR is the following;

$$SPR = \frac{EPR_{fished}}{EPR_{nofished}}$$

where;

$$EPR_{fished} = \sum_a \begin{cases} E_a, a = 0 \\ e^{-Z_{a-1}a} E_a, 0 < a < a_{\leq} max \end{cases}$$

where $Z_a = M + F_a$, and E is egg production at age assumed to be proportional to weight;

$$E_a \in Mat_a W_a$$

on the other hand, the calculation of the reproductive potential is the same as that of those captured without F;

$$EPR_{nofished} = \sum_a E_a e^{-M_a}$$

Assuming that egg production is proportional to the size of mature fish, relative fecundity-at-size is given by;

$$Fec_{L,g} = Mat_{L,g} L^b$$

where b is value of the exponent to reflect differente size fecunditity relationship and g is the fraction of recruits to group.

Assuming reasonable estimates of the M/K ratio, L_∞ (or CVL_∞), size-at-maturity, the parameters F/M, SL_{50}, and SL_{95} can be estimated from a representative sample of the length structure of the catch by minimizing the following multinomial negative loglikelihood function (NLL):

$$NLL = argmin \sum_i O_i ln \frac{\hat{P}_i}{\hat{O}_i}$$

where O_i and $\hat{O}_i$ are the observed number and proportion in length class i, respectively, and $\hat{P}_i$ is the model estimate of the probability in length class i (A. R. Hordyk et al., 2016).

The LBSPR package can be used to generate the expected size composition, the SPR, and relative yield for a given set of biological and exploitation pattern parameters. The output of the `LBSPRsim` function is an object of class `LB_obj`. This is another LBSPR object, and contains all of the information from the `LB_pars` object and the output of the `LBSPRsim` function.

It is important to note that the F/M ratio reported in the LBSPR model refers to the apical F over the adult natural mortality rate. That is, the value for fishing mortality refers to the highest level of F experienced by any single size class (A. Hordyk et al., 2014). If the selectivity pattern excludes all but the largest individuals from being exploited, it is possible to have a very high F/M ratio in a sustainable fishery (high SPR) and visceverse. Note that only the life history parameters need to be specified for the estimation model. The exploitation parameters will be estimated (A. Hordyk et al., 2014).

Two objects are required to fit the LBSPR model to length data: life-history parameters (`LB_pars`) described previously (2.4. section) and size compositions (`LB_lengths`), which contains the length frequency data. We provide a set of global size data and also by strata, with which we will be able to identify the spatial differences of the potential.

```
MyLengths <- new("LB_lengths")
slotNames(MyLengths)
```

```
## [1] "LMids"   "LData"   "L_units" "Years"   "NYears"  "Elog"
```

However, it is probably easier to create the `LB_lengths` object by directly reading in a `.csv`. A length frequency data of krill set with multiple years (2001-2020) by strata in 48.1 Subarea.

```r
# Brainsflied Strata
datdir <- setwd("~/DOCAS/LBSPR_Krill")
Lenbs <- new("LB_lengths", LB_pars = MyPars, file = paste0(datdir,
    "/lenghtBS_nochina.csv"), dataType = "freq", sep = ",", header = T)
# Elephan Island Strata
Lenei <- new("LB_lengths", LB_pars = MyPars, file = paste0(datdir,
    "/lenghtEI_nochina.csv"), dataType = "freq", sep = ",", header = T)
# Gerlache Strata
Lengc <- new("LB_lengths", LB_pars = MyPars, file = paste0(datdir,
    "/lenghtExtra_nochina.csv"), dataType = "freq", sep = ",",
    header = T)
# Join Strata
Lenjo <- new("LB_lengths", LB_pars = MyPars, file = paste0(datdir,
    "/lenghtJOIN_nochina.csv"), dataType = "freq", sep = ",",
    header = T)
# SSIW Strata
Lenssiw <- new("LB_lengths", LB_pars = MyPars, file = paste0(datdir,
    "/lenghtSSIW_nochina.csv"), dataType = "freq", sep = ",",
    header = T)
```

Fit the Model by strata

The LBSPR model is fitted by strata using the `LBSPRfit` function.

```r
fitbs <- LBSPRfit(MyPars, Lenbs)
fitei <- LBSPRfit(MyPars, Lenei)
fitgc <- LBSPRfit(MyPars, Lengc)
fitjo <- LBSPRfit(MyPars, Lenjo)
fitssiw <- LBSPRfit(MyPars, Lenssiw)
```

Sensitivity and perfomance analysis metohodology.

Ten sensitivity scenarios based on the upper and lower range for the asymptotic length von Bertalanffy L_∞ (55 to 65 mm) used in the base model (60 mm) were tested to identify the impact of this parameter on the SPR estimation, given that it is the parameters exhibiting the high degree of variability and one of the factors that most determines the estimates. On the other hand, the interdependence between krill and their environment is a well-known and influential factor in population dynamics, ecosystem impacts, and fishery. This interdependence also affects the reproductive potential and consequently, to any management decision that takes into account population parameters of krill. To assess the impact of individual growth variability, three scenarios of the VB k parameter were tested, representing different growth types (low = 0.2, medium = 0.7, and high = 1.2). Figure 2 displays a theoretical growth curve for krill based on three scenarios that were tested using LBSPR.

```r
# Definir los parámetros del modelo de Von Bertalanffy
L_inf <- 60
k <- c(0.2, 0.7, 1.2)
t_0 <- 0
# Crear un vector de edades
edades <- seq(0, 8, by = 0.2)
# Calcular los valores teóricos de crecimiento somático
df <- data.frame(edades = rep(edades, length(k)), k = rep(k,
    each = length(edades)))

df <- df %>%
    mutate(crecimiento = L_inf * (1 - exp(-k * (edades - t_0))))
```

```r
# Graficar la curva de crecimiento somático
sen <- ggplot(df, aes(x = edades, y = crecimiento, color = factor(k))) +
    geom_line(size = 1.5) + xlab("Ages") + ylab("Length (mm)") +
    ggtitle("") + theme(panel.background = element_rect(), panel.grid.major = element_blank(),
    panel.grid.minor = element_blank()) + scale_color_viridis_d(option = "C",
    name = "Theoretical growth", labels = c("Low", "Medium",
        "High")) + theme_few()
sen
```

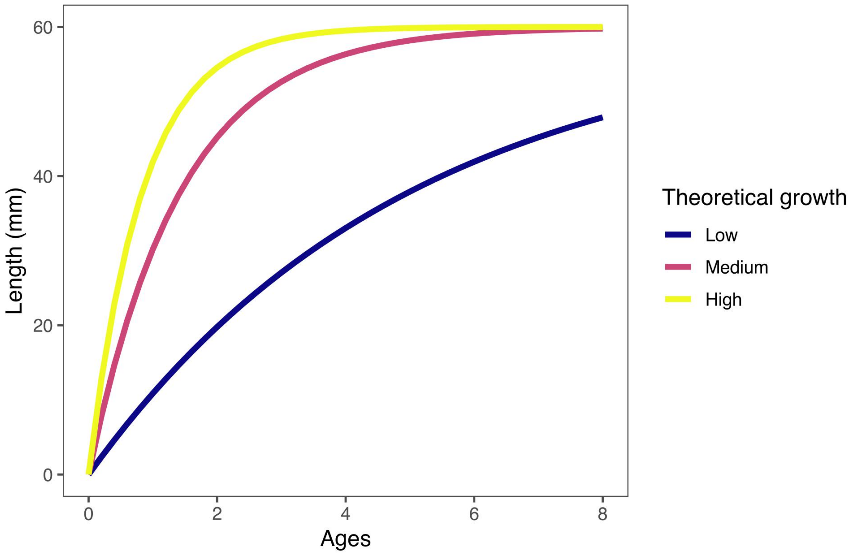

Figure 2: Theoretical growth curves for krill based on three SPR sensitivity scenarios that were tested using LBSPR.

After applying each scenario using each of the parameter setting, the results of scenarios are compared with the results provided by the methods based setting (Table 1), analyzing in this way the efect of underestimation/overestimation of the parameters L_∞ and k.

RESULTS

Model Perfomance

The results demonstrate good fits of the size structure distribution model for krill across the strata. The model accurately captures the distribution patterns of size classes, indicating its effectiveness in characterizing the population structure. The model successfully captures the variations in size distribution, reflecting the natural variability in krill populations across different strata. These findings suggest that the model can be utilized as a valuable tool for understanding and predicting size structure dynamics in krill populations (Figure 3).

```
bscom1 <- plotSize(fitbs, scales = "fixed", Title = "Bransfield strata") +
    thememau
eicom1 <- plotSize(fitei, Title = "Elephan Island strata") +
    thememau
gscom1 <- plotSize(fitgc, Title = "Gerlache strata") + thememau
jocom1 <- plotSize(fitjo, Title = "Joinville strata") + thememau
sswicom1 <- plotSize(fitssiw, Title = "SSWI strata") + thememau


ggarrange(bscom1, eicom1, gscom1, jocom1, sswicom1, ncol = 1,
    legend = "bottom", common.legend = TRUE)
```

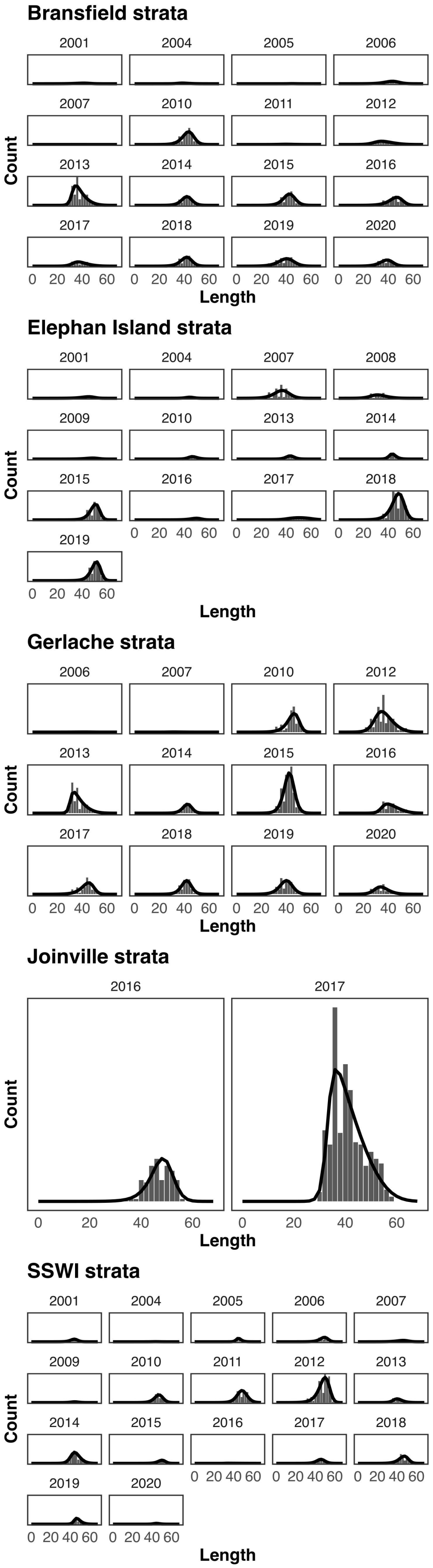

Figure 3: Fit of the model to the data of lengths in all strata

The difference between the observed accumulated size compositions for each stratum and compare it with the expected size composition at a target SPR (75% SPR). In the simulation of the structure in its virgin condition (without fishing), the red bars represent each stratum. Additionally, the overlap with the average structures observed during the years of fishery monitoring can be visualized. The SSWI Gerlache and EI strata exhibit the greatest differences from the simulated structure, possibly due to the significant contribution of juveniles in these strata. Conversely, the BS and JO strata demonstrate the closest resemblance to the simulated structure (Figure 4).

```
yr <- 5  # first year of data

bscom <- plotTarg(MyPars, Lenbs, yr = yr, Cols = c(2, 4), size.axtex = 8,
    title = "BS", targtext = FALSE) + thememau
eicom <- plotTarg(MyPars, Lenei, yr = yr, Cols = c(2, 4), size.axtex = 8,
    title = "EI", targtext = FALSE) + thememau
gccom <- plotTarg(MyPars, Lengc, yr = yr, Cols = c(2, 4), size.axtex = 8,
    title = "GS", targtext = FALSE) + thememau
jocom <- plotTarg(MyPars, Lenjo, yr = 1, Cols = c(2, 4), size.axtex = 8,
    title = "JOIN", targtext = FALSE) + thememau
sscom <- plotTarg(MyPars, Lenssiw, yr = yr, Cols = c(2, 4), size.axtex = 8,
    title = "SSWI", targtext = FALSE) + thememau

ggarrange(bscom, eicom + rremove("ylab"), gccom + rremove("ylab"),
    jocom, sscom + rremove("ylab"), ncol = 3, nrow = 2, legend = "bottom",
    common.legend = TRUE)
```

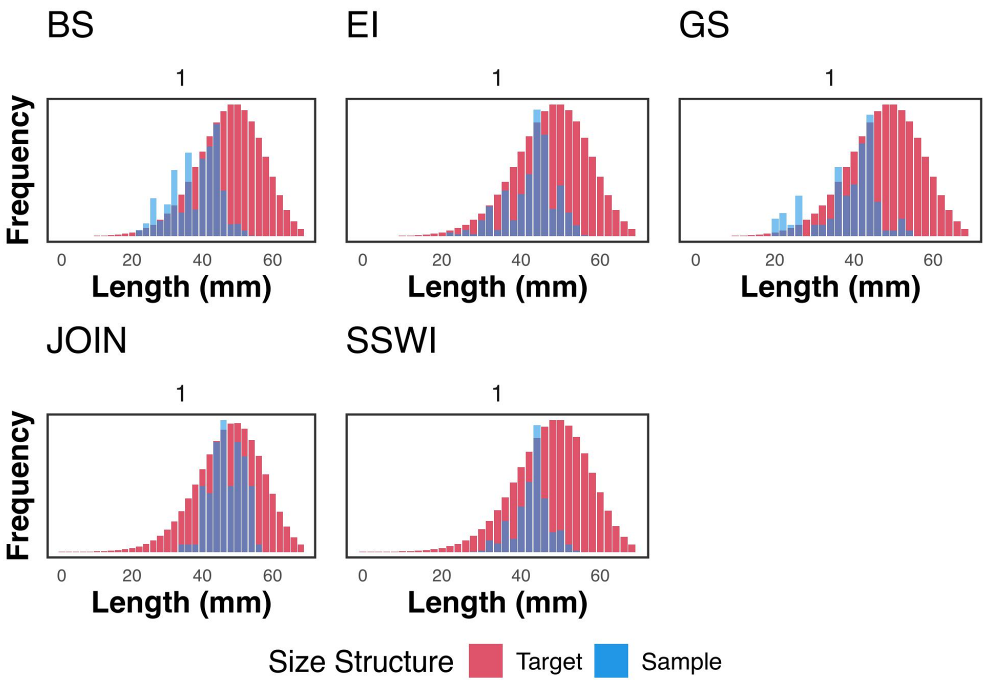

Figure 4: Difference between the observed accumulated size structure for each stratum related SPR objective

Furthermore, ogive maturity curve specific to each stratum and year, as well as the estimated length selectivity curve, are presented in (Figure 5). These curves offer crucial insights into the fishery's impact on the population and its reproductive status, providing measures of the population's vulnerability to fishing mortality. Notably, the Brainsfield stratum stands out with a lower proportion of mature individuals,

suggesting a higher prevalence of juveniles. It is important to note that the same maturity parameters were applied across all strata.

```
bsmat <- plotMat(fitbs, useSmooth = TRUE, Title = "BS") + thememau
eimat <- plotMat(fitei, useSmooth = TRUE, Title = "EI") + thememau
gcmat <- plotMat(fitgc, useSmooth = TRUE, Title = "GS") + thememau
jomat <- plotMat(fitjo, useSmooth = TRUE, Title = "JOIN") + thememau
ssmat <- plotMat(fitssiw, useSmooth = TRUE, Title = "SSWI") +
    thememau

ggarrange(bsmat, eimat + rremove("ylab"), gcmat, jomat + rremove("ylab"),
    ssmat, ncol = 2, nrow = 3, legend = "right", common.legend = TRUE)
```

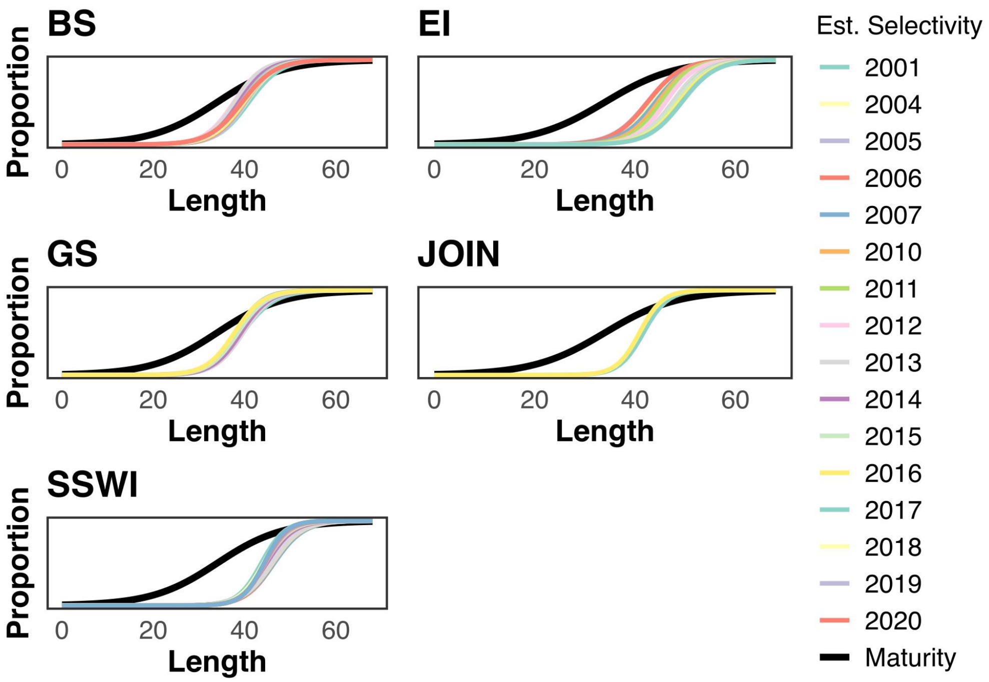

Figure 5: Maturity curves by strata

Comparing producivity between years and Stratas

The analysis of the krill population's reproductive potential across different years and strata reveals significant differences. Brainsfield and Gerlache strata exhibit a low reproductive potential below the proposed management target of 75% in the last year, with values of 0.121 and 0.085, respectively, falling even below the limit reference point. This condition arises from the concentration of a substantial number of immature individuals (juveniles) in these strata, which are being exploited by the fishery, thereby hindering their reproductive cycles from completing. On the contrary, the Elephant Island stratum demonstrates higher spawning potential ratio (SPR) levels in recent years, reaching 0.421 in 2019, which aligns closer to the management objective. This discrepancy is attributed to the spatial distribution of krill, as the Elephant Island stratum possesses a larger proportion of adult individuals compared to other strata.

```
sprbs <- as.data.frame(cbind(fitbs@Years, fitbs@SPR, fitbs@Vars[,
    4]))
colnames(sprbs) <- c("Year", "SPR", "Var")
sprbs$SPRv <- rep("BS", nrow(sprbs))
```

```
sprei <- as.data.frame(cbind(fitei@Years, fitei@SPR, fitei@Vars[,
    4]))
colnames(sprei) <- c("Year", "SPR", "Var")
sprei$SPRv <- rep("EI", nrow(sprei))

sprgc <- as.data.frame(cbind(fitgc@Years, fitgc@SPR, fitgc@Vars[,
    4]))
colnames(sprgc) <- c("Year", "SPR", "Var")
sprgc$SPRv <- rep("GS", nrow(sprgc))

sprjo <- as.data.frame(cbind(fitjo@Years, fitjo@SPR, fitjo@Vars[,
    4]))
colnames(sprjo) <- c("Year", "SPR", "Var")
sprjo$SPRv <- rep("JOIN", nrow(sprjo))

sprssiw <- as.data.frame(cbind(fitssiw@Years, fitssiw@SPR, fitssiw@Vars[,
    4]))
colnames(sprssiw) <- c("Year", "SPR", "Var")
sprssiw$SPRv <- rep("SSWI", nrow(sprssiw))

allspr <- rbind(sprbs, sprei, sprgc, sprjo, sprssiw)

allspr <- allspr %>%
    mutate(SD = sqrt(Var))


allsprwide <- round(pivot_wider(allspr, names_from = SPRv, values_from = c(SPR,
    Var, SD), values_fill = NA, names_sep = " ", ), 3)
```

Figure 6 provides a visual representation of the SPR trends across years and strata, clearly indicating the references (yellow line = 75% SPR Objective and Red line = 20% Limit SPR).

```
allsprpl2 <- ggplot(allspr, aes(Year, SPR)) + geom_point(alpha = 0.8) +
    geom_errorbar(aes(ymin = SPR - SD, ymax = SPR + SD), width = 0.2,
        alpha = 0.5) + geom_smooth(method = "lm", alpha = 0.3,
    se = TRUE, col = "black", level = 0.75) + geom_hline(yintercept = 0.75,
    colour = "#006d2c", alpha = 0.5, linewidth = 1.2, linetype = 2) +
    geom_hline(yintercept = 0.2, colour = "#bd0026", alpha = 0.5,
        linewidth = 1.2) + facet_wrap(. ~ SPRv, ncol = 5) + xlim(2001,
    2021) + ylim(0, 0.9) + theme_few() + theme(legend.position = "none",
    axis.text.x = element_text(angle = 90, hjust = 1), panel.background = element_rect(fill = "white"),
    panel.grid.major = element_blank(), panel.grid.minor = element_blank())

allsprpl2
```

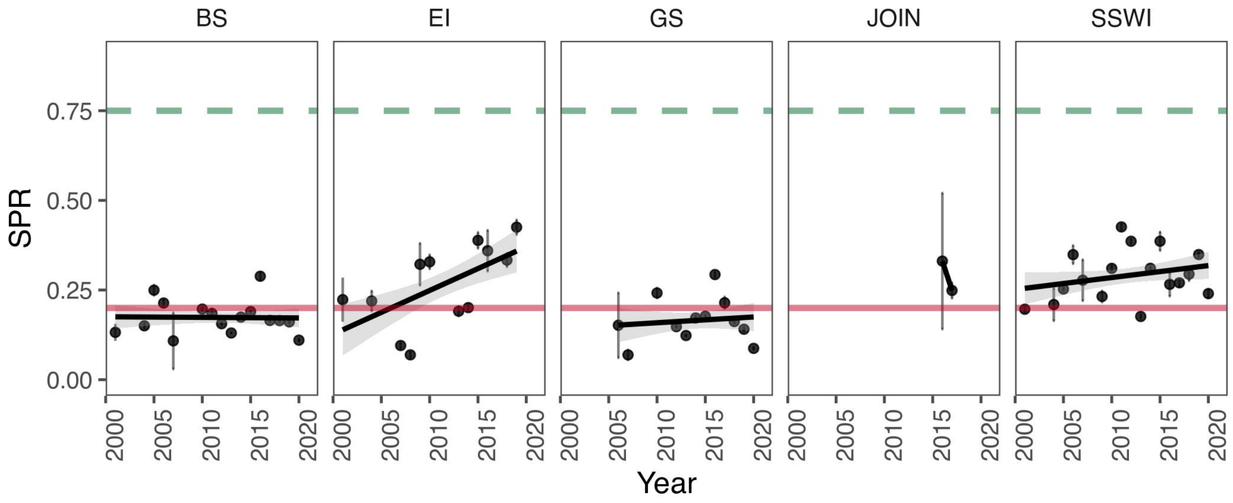

Figure 6: Krill Intrinsic Productivity (SPR) by strata and by year

All the estimated values of SPR and their associated variance by stratum and by year can be identified in Table 2.

Table 2: Estimates of SPR by Strata (values in parentheses represent the standard deviation)

Year	BS	EI	GS	JOIN	SSWI
2001	0.132 (0.024)	0.223 (0.062)	NA	NA	0.196 (0.011)
2004	0.15 (0.008)	0.219 (0.028)	NA	NA	0.21 (0.045)
2005	0.25 (0.014)	NA	NA	NA	0.253 (0.005)
2006	0.214 (0.009)	NA	0.152 (0.09)	NA	0.349 (0.025)
2007	0.108 (0.078)	0.095 (0.008)	0.069 (0.014)	NA	0.277 (0.057)
2008	NA	0.069 (0.008)	NA	NA	NA
2009	NA	0.322 (0.058)	NA	NA	0.232 (0.016)
2010	0.197 (0.004)	0.329 (0.02)	0.242 (0.014)	NA	0.31 (0.008)
2011	0.185 (0.012)	NA	NA	NA	0.426 (0.009)
2012	0.156 (0.004)	NA	0.148 (0.003)	NA	0.386 (0.006)
2013	0.13 (0.001)	0.191 (0.013)	0.123 (0.002)	NA	0.176 (0.005)
2014	0.175 (0.002)	0.201 (0.007)	0.172 (0.006)	NA	0.311 (0.004)
2015	0.19 (0.003)	0.389 (0.022)	0.177 (0.003)	NA	0.386 (0.026)
2016	0.288 (0.01)	0.36 (0.057)	0.293 (0.007)	0.331 (0.19)	0.266 (0.033)
2017	0.165 (0.004)	1 (0)	0.214 (0.016)	0.249 (0.022)	0.27 (0.01)
2018	0.165 (0.003)	0.334 (0.019)	0.162 (0.004)	NA	0.294 (0.018)
2019	0.161 (0.004)	0.425 (0.02)	0.141 (0.005)	NA	0.349 (0.006)
2020	0.11 (0.004)	NA	0.087 (0.004)	NA	0.24 (0.012)

Sensitivity and perfomance analysis

The results of the methods in the reference setting are compared to the obtained under overestimation/underestimation in intrinsic productivity regarding asymptotic length von Bertalanffy L_∞ parameter in krill. First, it was possible to identify that for all strata, the impact of low L_∞ ranges under the base model (60 mm) overestimates the level of reproductive potential krill with values between 42% and 32% (Bransfield and Elephant Island strata respectively). Regarding higher L_∞ settings, the model tends to underestimate the reproductive potential with values between -25% and -30% (Gerlache and Joinville strata) Figure 7, Table **??**.

```
for (i in 55:65) {
    nombre_objeto <- paste0("MyPars_", i)
    objeto_modificado <- MyPars  # Crear una copia del objeto original

    objeto_modificado@Linf <- i  # Modificar el valor de @Linf en cada iteración

    assign(nombre_objeto, objeto_modificado, envir = .GlobalEnv)

    # Resto del código que deseas ejecutar con el objeto
    # modificado A blank LB_pars object created Default
    # values have been set for some parameters
    MyPars@Species <- "Euphausia superba"
    MyPars@L50 <- 34
    MyPars@L95 <- 55  # verrificar bibliografia
    MyPars@MK <- 0.4/0.45

    # Explotation
    MyPars@SL50 <- 40  #numeric() #1
    MyPars@SL95 <- 56  #numeric() #27
    MyPars@SPR <- 0.75  #numeric()# ###cambia el numero 0.4 a en blanco
    MyPars@BinWidth <- 1
    # MyPars@FM <- 1

    MyPars@Walpha <- 1
    MyPars@Wbeta <- 3.0637  #r2 = 0.9651

    MyPars@BinWidth <- 1
    MyPars@BinMax <- 70
    MyPars@BinMin <- 0
    MyPars@L_units <- "mm"

    # Imprimir el valor de @Linf después de cada cambio
    # print(get(nombre_objeto)@Linf)
}
```

```
# ciclo de ajuste para BS
for (i in 55:65) {
    nombre_objeto <- paste0("MyPars_", i)  # Nombre del objeto S4 en cada iteración
    nombre_variable <- paste0("fitbs", i)  # Nuevo nombre para el resultado

    # Obtener el objeto S4 correspondiente
    objeto <- get(nombre_objeto)

    # Ejecutar LBSPRfit() y asignar el resultado a una
    # nueva variable con nombre único
```

```
    assign(nombre_variable, LBSPRfit(objeto, Lenbs), envir = .GlobalEnv)
}


# ciclo de ajuste para Gerlache
for (i in 55:65) {
    nombre_objeto <- paste0("MyPars_", i)
    nombre_variable <- paste0("fitei", i)
    objeto <- get(nombre_objeto)
    assign(nombre_variable, LBSPRfit(objeto, Lenei), envir = .GlobalEnv)
}

# ciclo de ajuste para Gerlache
for (i in 55:65) {
    nombre_objeto <- paste0("MyPars_", i)
    nombre_variable <- paste0("fitgc", i)
    objeto <- get(nombre_objeto)
    assign(nombre_variable, LBSPRfit(objeto, Lengc), envir = .GlobalEnv)
}

# ciclo de ajuste para JoinVIlle
for (i in 55:65) {
    nombre_objeto <- paste0("MyPars_", i)
    nombre_variable <- paste0("fitjo", i)
    objeto <- get(nombre_objeto)
    assign(nombre_variable, LBSPRfit(objeto, Lenjo), envir = .GlobalEnv)
}

# ciclo de ajuste para JSSWI
for (i in 55:65) {
    nombre_objeto <- paste0("MyPars_", i)
    nombre_variable <- paste0("fitsswi", i)
    objeto <- get(nombre_objeto)
    assign(nombre_variable, LBSPRfit(objeto, Lenssiw), envir = .GlobalEnv)
}
```

```
# Genero lo que quiero comparar BS
valbs <- as.data.frame(cbind(fitbs55@Years, fitbs55@SPR, fitbs56@SPR,
    fitbs57@SPR, fitbs58@SPR, fitbs59@SPR, fitbs60@SPR, fitbs61@SPR,
    fitbs62@SPR, fitbs63@SPR, fitbs64@SPR, fitbs65@SPR))
colnames(valbs) <- c("Years", "Linf55", "Linf56", "Linf57", "Linf58",
    "Linf59", "Linf60", "Linf61", "Linf62", "Linf63", "Linf64",
    "Linf65")

valbs_largo <- pivot_longer(valbs, cols = c(2:12, ), names_to = "Parameter",
    values_to = "SPR")
valbs_largo$SPRstra <- rep("BS", nrow(valbs_largo))

# Genero lo que quiero comparar EI
valei <- as.data.frame(cbind(fitei55@Years, fitei55@SPR, fitei56@SPR,
    fitei57@SPR, fitei58@SPR, fitei59@SPR, fitei60@SPR, fitei61@SPR,
    fitei62@SPR, fitei63@SPR, fitei64@SPR, fitei65@SPR))
colnames(valei) <- c("Years", "Linf55", "Linf56", "Linf57", "Linf58",
    "Linf59", "Linf60", "Linf61", "Linf62", "Linf63", "Linf64",
```

```
    "Linf65")

valei_largo <- pivot_longer(valei, cols = c(2:12, ), names_to = "Parameter",
    values_to = "SPR")
valei_largo$SPRstra <- rep("EI", nrow(valei_largo))
# Genero lo que quiero comparar Gerlache
valgc <- as.data.frame(cbind(fitgc55@Years, fitgc55@SPR, fitgc56@SPR,
    fitgc57@SPR, fitgc58@SPR, fitgc59@SPR, fitgc60@SPR, fitgc61@SPR,
    fitgc62@SPR, fitgc63@SPR, fitgc64@SPR, fitgc65@SPR))
colnames(valgc) <- c("Years", "Linf55", "Linf56", "Linf57", "Linf58",
    "Linf59", "Linf60", "Linf61", "Linf62", "Linf63", "Linf64",
    "Linf65")

valgc_largo <- pivot_longer(valgc, cols = c(2:12, ), names_to = "Parameter",
    values_to = "SPR")
valgc_largo$SPRstra <- rep("GS", nrow(valgc_largo))

# Genero lo que quiero comparar JOin
valjo <- as.data.frame(cbind(fitjo55@Years, fitjo55@SPR, fitjo56@SPR,
    fitjo57@SPR, fitjo58@SPR, fitjo59@SPR, fitjo60@SPR, fitjo61@SPR,
    fitjo62@SPR, fitjo63@SPR, fitjo64@SPR, fitjo65@SPR))
colnames(valjo) <- c("Years", "Linf55", "Linf56", "Linf57", "Linf58",
    "Linf59", "Linf60", "Linf61", "Linf62", "Linf63", "Linf64",
    "Linf65")

valjo_largo <- pivot_longer(valjo, cols = c(2:12, ), names_to = "Parameter",
    values_to = "SPR")
valjo_largo$SPRstra <- rep("JOIN", nrow(valjo_largo))

# Genero lo que quiero comparar SSWI
valsswi <- as.data.frame(cbind(fitsswi55@Years, fitsswi55@SPR,
    fitsswi56@SPR, fitsswi57@SPR, fitsswi58@SPR, fitsswi59@SPR,
    fitsswi60@SPR, fitsswi61@SPR, fitsswi62@SPR, fitsswi63@SPR,
    fitsswi64@SPR, fitsswi65@SPR))
colnames(valsswi) <- c("Years", "Linf55", "Linf56", "Linf57",
    "Linf58", "Linf59", "Linf60", "Linf61", "Linf62", "Linf63",
    "Linf64", "Linf65")

valsswi_largo <- pivot_longer(valsswi, cols = c(2:12, ), names_to = "Parameter",
    values_to = "SPR")
valsswi_largo$SPRstra <- rep("SSWI", nrow(valsswi_largo))
# agrupo
valtodo <- rbind(valsswi_largo, valjo_largo, valbs_largo, valei_largo,
    valgc_largo)
```

Figure 7 provides a plot by strata with scenarios of L_{inf} of the SPR trends across strata (yellow line = 75% SPR Objective and Red line = 20% Limit SPR).

```
valtodo <- valtodo %>%
    mutate(Highlight = ifelse(Parameter == "Linf60", "Highlighted",
        "Normal"))
sensproto <- ggplot(valtodo %>%
    drop_na() %>%
    filter(SPR < 0.65), aes(x = SPR, y = Parameter)) + geom_boxplot(aes(fill = ifelse(Parameter ==
```

```
    "Linf60", "red", NA)), trim = TRUE, color = "black", position = position_dodge(0.9),
    adjust = 1/5) + geom_vline(xintercept = 0.75, colour = "#006d2c",
    alpha = 0.5, linetype = 2) + geom_vline(xintercept = 0.2,
    colour = "#bd0026", alpha = 0.5) + geom_jitter(aes(fill = ifelse(Parameter ==
    "Linf60", "red", NA)), shape = 21, color = "black", alpha = 0.5,
    height = 0, width = 0.1) + facet_wrap(~SPRstra, ncol = 11) +
    labs(x = "SPR", y = "VB Parameters tested") + theme_minimal() +
    theme(legend.position = "none", axis.text.x = element_text(angle = 90,
        hjust = 2), panel.background = element_rect(), panel.grid.major = element_blank(),
        panel.grid.minor = element_blank()) + xlim(0, 1) + scale_fill_identity()

sensproto
```

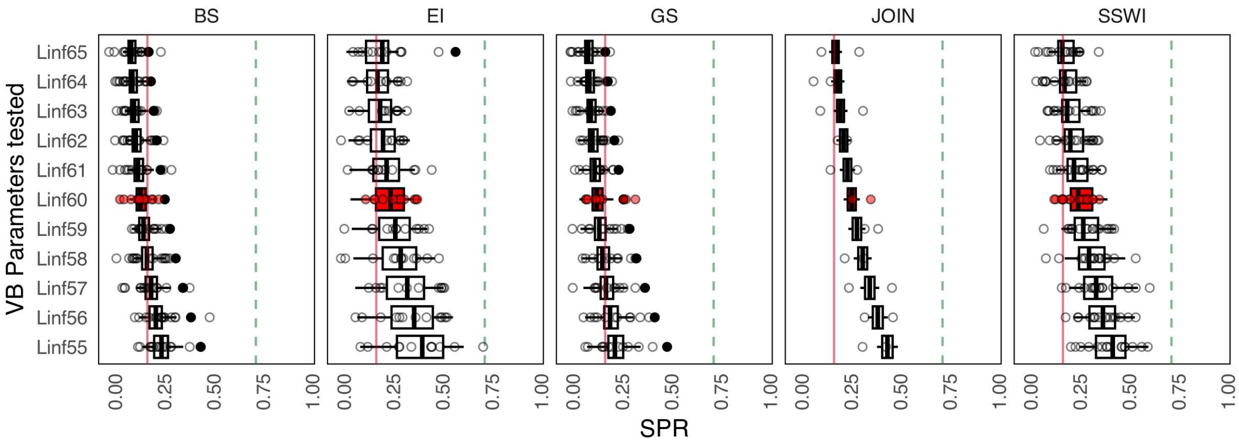

Figure 7: Sensitivity analysis by strata about asymptotic length VB

```
tablinf <- valtodo %>%
    group_by(Parameter, SPRstra) %>%
    summarise(Median = mean(SPR), Variance = sd(SPR)) %>%
    pivot_wider(names_from = SPRstra, values_from = c(Median,
        Variance), names_sep = " ") %>%
    rename(`VB scenario` = "Parameter") %>%
    mutate_if(is.numeric, round, 2)



tablinf_reorganizado <- tablinf %>%
    mutate(BS = paste0(`Median BS`, " (", `Variance BS`, ")"),
        EI = paste0(`Median EI`, " (", `Variance EI`, ")"), GS = paste0(`Median GS`,
            " (", `Variance GS`, ")"), JOIN = paste0(`Median JOIN`,
            " (", `Variance JOIN`, ")"), SSWI = paste0(`Median SSWI`,
            " (", `Variance SSWI`, ")")) %>%
    select(`VB scenario`, BS, EI, GS, JOIN, SSWI)

# write_csv(tablinf_reorganizado, 'SPR_Linf.csv')
```

All the estimated values of SPR by L_{inf}scenario by stratum can be identified in Table 3.

Regarding the three growth levels of the species (high, medium, low) referred to the parameter k, it was

Table 3: Estimated by asymptotyc lenght (VB) scenario (values in parentheses represent the standard deviation)

VB scenario	BS	EI	GS	JOIN	SSWI
Linf55	0.28 (0.07)	0.46 (0.23)	0.27 (0.11)	0.47 (0.07)	0.47 (0.13)
Linf56	0.25 (0.07)	0.42 (0.23)	0.24 (0.09)	0.42 (0.07)	0.42 (0.11)
Linf57	0.23 (0.06)	0.39 (0.23)	0.22 (0.08)	0.38 (0.07)	0.38 (0.1)
Linf58	0.21 (0.06)	0.36 (0.23)	0.2 (0.08)	0.35 (0.06)	0.34 (0.09)
Linf59	0.19 (0.05)	0.34 (0.23)	0.18 (0.07)	0.32 (0.06)	0.32 (0.08)
Linf60	0.17 (0.05)	0.32 (0.23)	0.17 (0.06)	0.29 (0.06)	0.29 (0.07)
Linf61	0.16 (0.04)	0.3 (0.21)	0.15 (0.06)	0.27 (0.06)	0.27 (0.07)
Linf62	0.15 (0.04)	0.27 (0.18)	0.14 (0.05)	0.25 (0.05)	0.25 (0.06)
Linf63	0.14 (0.04)	0.25 (0.17)	0.13 (0.05)	0.23 (0.05)	0.23 (0.06)
Linf64	0.13 (0.04)	0.24 (0.15)	0.13 (0.05)	0.22 (0.05)	0.22 (0.05)
Linf65	0.12 (0.04)	0.22 (0.14)	0.12 (0.04)	0.21 (0.05)	0.21 (0.05)

possible to identify that high and medium growth types result in very low SPR (spawning potential ratio) estimates compared to slow and medium growth. In fact, with high individual growth, the model estimates that SPR levels would be very close to the target management levels (75% SPR) and far from the limit reference level of 20% (Figure 8, Table 4).

```
for (i in c(2, 0.5, 0.3)) {
    nombre_objeto <- paste0("MyPars_", i)  # Crear un nombre único para cada objeto modificado
    objeto_modificado <- MyPars  # Crear una copia del objeto original

    objeto_modificado@MK <- i  # Modificar el valor de @Linf en cada iteración

    assign(nombre_objeto, objeto_modificado, envir = .GlobalEnv)  # Asignar el objeto modificado al nom

    # Resto del código que deseas ejecutar con el objeto
    # modificado A blank LB_pars object created Default
    # values have been set for some parameters
    MyPars@Species <- "Euphausia superba"
    MyPars@L50 <- 34
    MyPars@L95 <- 55  # verrificar bibliografia
    MyPars@Linf <- 60

    # Explotation
    MyPars@SL50 <- 40  #numeric() #1
    MyPars@SL95 <- 56  #numeric() #27
    MyPars@SPR <- 0.75  #numeric()# ###cambia el numero 0.4 a en blanco
    MyPars@BinWidth <- 1
    # MyPars@FM <- 1

    MyPars@Walpha <- 1
    MyPars@Wbeta <- 3.0637  #r2 = 0.9651

    MyPars@BinWidth <- 1
    MyPars@BinMax <- 70
    MyPars@BinMin <- 0
    MyPars@L_units <- "mm"
```

```r
    # Imprimir el valor de @Linf después de cada cambio
    # print(get(nombre_objeto)@Linf)
}
```

```r
# ciclo de ajuste para BS
for (i in c(2, 0.5, 0.3)) {
    nombre_objeto <- paste0("MyPars_", i)  # Nombre del objeto S4 en cada iteración
    nombre_variable <- paste0("fitbs", i)  # Nuevo nombre para el resultado

    # Obtener el objeto S4 correspondiente
    objeto <- get(nombre_objeto)

    # Ejecutar LBSPRfit() y asignar el resultado a una
    # nueva variable con nombre único
    assign(nombre_variable, LBSPRfit(objeto, Lenbs), envir = .GlobalEnv)
}


# ciclo de ajuste para Gerlache
for (i in c(2, 0.5, 0.3)) {
    nombre_objeto <- paste0("MyPars_", i)
    nombre_variable <- paste0("fitei", i)
    objeto <- get(nombre_objeto)
    assign(nombre_variable, LBSPRfit(objeto, Lenei), envir = .GlobalEnv)
}

# ciclo de ajuste para Gerlache
for (i in c(2, 0.5, 0.3)) {
    nombre_objeto <- paste0("MyPars_", i)
    nombre_variable <- paste0("fitgc", i)
    objeto <- get(nombre_objeto)
    assign(nombre_variable, LBSPRfit(objeto, Lengc), envir = .GlobalEnv)
}

# ciclo de ajuste para JoinVIlle
for (i in c(2, 0.5, 0.3)) {
    nombre_objeto <- paste0("MyPars_", i)
    nombre_variable <- paste0("fitjo", i)
    objeto <- get(nombre_objeto)
    assign(nombre_variable, LBSPRfit(objeto, Lenjo), envir = .GlobalEnv)
}

# ciclo de ajuste para JSSWI
for (i in c(2, 0.5, 0.3)) {
    nombre_objeto <- paste0("MyPars_", i)
    nombre_variable <- paste0("fitsswi", i)
    objeto <- get(nombre_objeto)
    assign(nombre_variable, LBSPRfit(objeto, Lenssiw), envir = .GlobalEnv)
}
```

```r
# Genero lo que quiero comparar BS
valprobs <- as.data.frame(cbind(fitbs0.3@Years, fitbs0.3@SPR,
    fitbs0.5@SPR, fitbs2@SPR))
colnames(valprobs) <- c("Years", "High", "Med", "Low")
```

```r
valprobs_largo <- pivot_longer(valprobs, cols = c(2:4, ), names_to = "Parameter",
    values_to = "SPR")
valprobs_largo$SPRstra <- rep("BS", nrow(valprobs_largo))

# Genero lo que quiero comparar EI
valproei <- as.data.frame(cbind(fitei0.3@Years, fitei0.3@SPR,
    fitei0.5@SPR, fitei2@SPR))
colnames(valproei) <- c("Years", "High", "Med", "Low")

valproei_largo <- pivot_longer(valproei, cols = c(2:4, ), names_to = "Parameter",
    values_to = "SPR")
valproei_largo$SPRstra <- rep("EI", nrow(valproei_largo))
# Genero lo que quiero comparar Gerlache
valprogc <- as.data.frame(cbind(fitgc0.3@Years, fitgc0.3@SPR,
    fitgc0.5@SPR, fitgc2@SPR))
colnames(valprogc) <- c("Years", "High", "Med", "Low")

valprogc_largo <- pivot_longer(valprogc, cols = c(2:4, ), names_to = "Parameter",
    values_to = "SPR")
valprogc_largo$SPRstra <- rep("GS", nrow(valprogc_largo))
# Genero lo que quiero comparar JOin
valprojo <- as.data.frame(cbind(fitjo0.3@Years, fitjo0.3@SPR,
    fitjo0.5@SPR, fitjo2@SPR))
colnames(valprojo) <- c("Years", "High", "Med", "Low")

valprojo_largo <- pivot_longer(valprojo, cols = c(2:4, ), names_to = "Parameter",
    values_to = "SPR")
valprojo_largo$SPRstra <- rep("JOIN", nrow(valprojo_largo))

# Genero lo que quiero comparar SSWI
valprosswi <- as.data.frame(cbind(fitsswi0.3@Years, fitsswi0.3@SPR,
    fitsswi0.5@SPR, fitsswi2@SPR))
colnames(valprosswi) <- c("Years", "High", "Med", "Low")

valprosswi_largo <- pivot_longer(valprosswi, cols = c(2:4, ),
    names_to = "Parameter", values_to = "SPR")
valprosswi_largo$SPRstra <- rep("SSWI", nrow(valprosswi_largo))
# agrupo
valprotodo <- rbind(valprosswi_largo, valproei_largo, valprobs_largo,
    valprojo_largo, valprogc_largo)
```

Table 4: Estimates of SPR by Growth Scenario (values in parentheses represent the standard deviation)

Growth scenario	BS	EI	GS	JOIN	SSWI
High	0.05 (0.02)	0.1 (0.08)	0.04 (0.02)	0.08 (0.03)	0.09 (0.03)
Low	0.49 (0.08)	0.61 (0.19)	0.47 (0.14)	0.71 (0.05)	0.65 (0.11)
Med	0.08 (0.03)	0.17 (0.13)	0.08 (0.03)	0.15 (0.05)	0.15 (0.05)

```r
sensproto2 <- ggplot(valprotodo %>%
    filter(SPR < 0.99) %>%
    drop_na() %>%
```

```
    mutate(Parameter = factor(Parameter, levels = c("Low", "Med",
        "High"))), aes(x = Parameter, y = SPR)) + geom_boxplot(aes(fill = Parameter),
    alpha = 0.6) + geom_hline(yintercept = 0.75, colour = "#006d2c",
    alpha = 0.5, linetype = "dashed") + geom_hline(yintercept = 0.2,
    colour = "#bd0026", alpha = 0.5) + geom_jitter(aes(color = Parameter),
    alpha = 0.5, width = 0.1) + facet_wrap(~SPRstra, ncol = 5) +
    labs(y = "SPR", x = "Growth Scenario") + theme_minimal() +
    theme(legend.position = "none", panel.background = element_rect(),
        panel.grid.major = element_blank(), panel.grid.minor = element_blank()) +
    scale_fill_manual(values = c(Low = "white", Med = "red",
        High = "white")) + scale_color_manual(values = c(Low = "black",
    Med = "red", High = "black"))


sensproto2
```

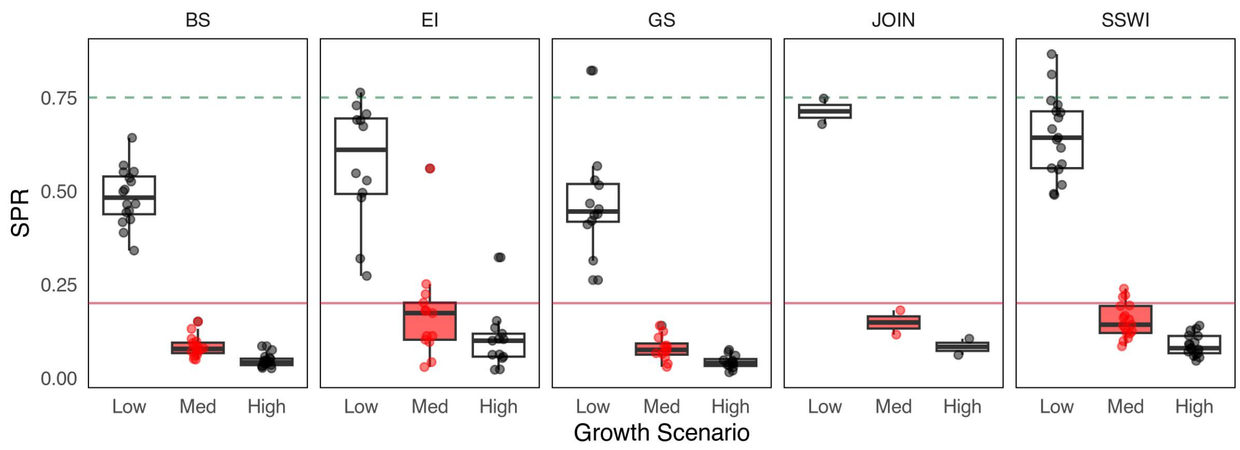

Figure 8: Sensitivity analysis by strata about krill growth type

Analysis to three Subareas (48.1, 48.2 and 48.3)

Reading data

```
datdir <- setwd("~/DOCAS/LBSPR_Krill")
Len481 <- new("LB_lengths", LB_pars = MyPars, file = paste0(datdir,
    "/lenght481_nochina.csv"), dataType = "freq", sep = ",",
    header = T)

Len482 <- new("LB_lengths", LB_pars = MyPars, file = paste0(datdir,
    "/lenght482_nochina.csv"), dataType = "freq", sep = ",",
    header = T)

Len483 <- new("LB_lengths", LB_pars = MyPars, file = paste0(datdir,
    "/lenght483_nochina.csv"), dataType = "freq", sep = ",",
    header = T)
```

The LBSPR model is fitted by strata using the `LBSPRfit` function.

```
fit481 <- LBSPRfit(MyPars, Len481)
fit482 <- LBSPRfit(MyPars, Len482)
fit483 <- LBSPRfit(MyPars, Len483)
```

Now, to paper propose, we estimate SPR to all subareas (48.1, 48.2 and 48.3)

```
# Get values estimated

spr481 <- as.data.frame(cbind(fit481@Years, fit481@SPR, fit481@Vars[,
    4]))
colnames(spr481) <- c("Year", "SPR", "Var")
spr481$SPRv <- rep("481", nrow(spr481))

spr482 <- as.data.frame(cbind(fit482@Years, fit482@SPR, fit482@Vars[,
    4]))
colnames(spr482) <- c("Year", "SPR", "Var")
spr482$SPRv <- rep("482", nrow(spr482))

spr483 <- as.data.frame(cbind(fit483@Years, fit483@SPR, fit483@Vars[,
    4]))
colnames(spr483) <- c("Year", "SPR", "Var")
spr483$SPRv <- rep("483", nrow(spr483))


spr48 <- rbind(spr481, spr482, spr483)

spr48 <- spr48 %>%
    mutate(SD = sqrt(Var))

spr48wide <- round(pivot_wider(spr48, names_from = SPRv, values_from = c(SPR,
    Var, SD), values_fill = NA, names_sep = " ", ), 3)
```

Figure 9 show the SPR trends across years to 48.1, 48.2 and 48.3, indicating the references (yellow line = 75% SPR Objective and Red line = 20% Limit SPR).

```
spr48plot <- ggplot(spr48, aes(Year, SPR)) + geom_point(alpha = 0.8) +
    geom_errorbar(aes(ymin = SPR - SD, ymax = SPR + SD), width = 0.2,
        alpha = 0.5) + geom_smooth(method = "lm", alpha = 0.3,
    se = TRUE, col = "black", level = 0.75) + geom_hline(yintercept = 0.75,
    colour = "#006d2c", alpha = 0.5, linetype = 2) + geom_hline(yintercept = 0.2,
    colour = "#bd0026", alpha = 0.5) + facet_wrap(. ~ SPRv, ncol = 5) +
    xlim(2001, 2021) + ylim(0, 0.9) + theme_few() + theme(legend.position = "none",
    axis.text.x = element_text(angle = 90, hjust = 1), panel.background = element_rect(fill = "white"),
    panel.grid.major = element_blank(), panel.grid.minor = element_blank())
spr48plot
```

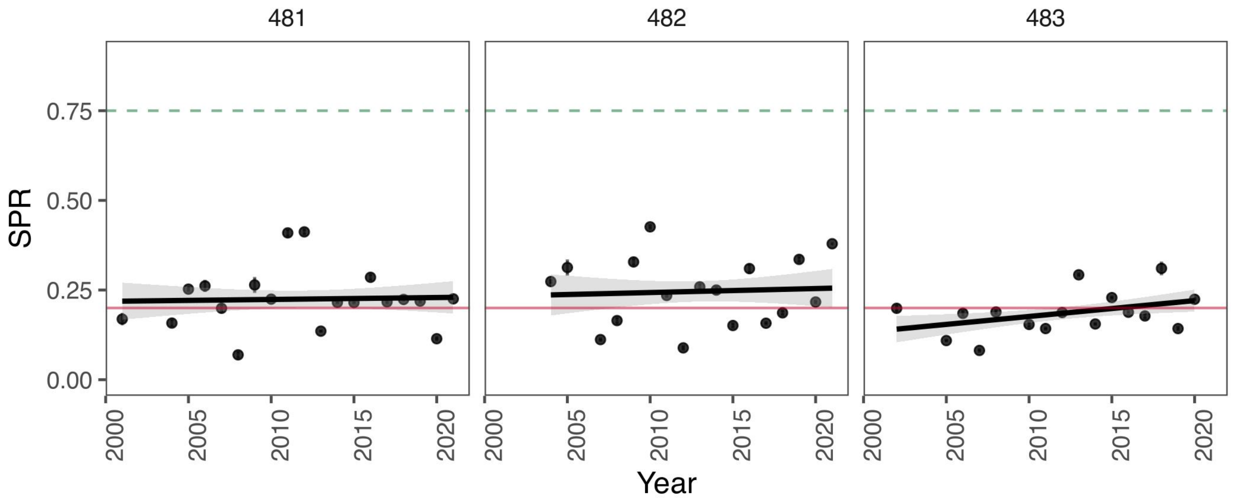

Figure 9: Krill Intrinsic Productivity (SPR) by subarea and by year

Main outputs in Table 5.

Table 5: Estimates of SPR by SubArea (parentheses represent the standard deviation)

Year	481	482	483
2001	0.169 (0.017)	NA	NA
2002	NA	NA	0.199 (0.008)
2004	0.158 (0.009)	0.274 (0.004)	NA
2005	0.253 (0.004)	0.313 (0.019)	0.109 (0.001)
2006	0.262 (0.013)	NA	0.185 (0.007)
2007	0.199 (0.01)	0.112 (0.003)	0.082 (0.003)
2008	0.069 (0.008)	0.165 (0.008)	0.189 (0.004)
2009	0.264 (0.019)	0.328 (0.009)	NA
2010	0.225 (0.003)	0.426 (0.004)	0.154 (0.007)
2011	0.409 (0.009)	0.235 (0.004)	0.143 (0.002)
2012	0.412 (0.006)	0.089 (0.004)	0.187 (0.002)
2013	0.135 (0.001)	0.259 (0.007)	0.292 (0.003)
2014	0.215 (0.002)	0.25 (0.009)	0.155 (0.002)
2015	0.215 (0.002)	0.151 (0.005)	0.229 (0.002)
2016	0.285 (0.008)	0.31 (0.011)	0.188 (0.003)
2017	0.217 (0.004)	0.158 (0.003)	0.178 (0.007)
2018	0.224 (0.002)	0.186 (0.006)	0.31 (0.016)
2019	0.219 (0.004)	0.335 (0.007)	0.143 (0.004)
2020	0.114 (0.003)	0.217 (0.003)	0.224 (0.002)
2021	0.225 (0.002)	0.379 (0.004)	NA
2022	0.876 (0.006)	0.271 (0.004)	0.244 (0.005)
2023	0.359 (0.003)	0.358 (0.006)	0.311 (0.004)

```
spr481plot <- ggplot(spr48 %>%
    filter(SPRv == "481"), aes(Year, SPR)) + geom_point(alpha = 0.8) +
    geom_errorbar(aes(ymin = SPR - SD, ymax = SPR + SD), width = 0.2,
```

```
        alpha = 0.5) + geom_smooth(method = "lm", alpha = 0.3,
    se = TRUE, col = "black", level = 0.75) + geom_hline(yintercept = 0.75,
    colour = "#006d2c", alpha = 0.5, linetype = 2) + geom_hline(yintercept = 0.2,
    colour = "#bd0026", alpha = 0.5) + xlim(2001, 2021) + ylim(0,
    0.9) + theme_few() + theme(legend.position = "none", axis.text.x = element_text(angle = 90,
    hjust = 1), panel.background = element_rect(fill = "white"),
    panel.grid.major = element_blank(), panel.grid.minor = element_blank())
```

sensitivity analysis to L_{inf} to 48.1

```
# ciclo de ajuste para 481
for (i in 55:65) {
    nombre_objeto <- paste0("MyPars_", i)
    nombre_variable <- paste0("fit481", i)
    objeto <- get(nombre_objeto)
    assign(nombre_variable, LBSPRfit(objeto, Len481), envir = .GlobalEnv)
}
```

```
# Genero lo que quiero comparar BS
val481 <- as.data.frame(cbind(fit48155@Years, fit48155@SPR, fit48156@SPR,
    fit48157@SPR, fit48158@SPR, fit48159@SPR, fit48160@SPR, fit48161@SPR,
    fit48162@SPR, fit48163@SPR, fit48164@SPR, fit48165@SPR))
colnames(val481) <- c("Years", "Linf55", "Linf56", "Linf57",
    "Linf58", "Linf59", "Linf60", "Linf61", "Linf62", "Linf63",
    "Linf64", "Linf65")

val481_largo <- pivot_longer(val481, cols = c(2:12, ), names_to = "Parameter",
    values_to = "SPR")
val481_largo$SPRstra <- rep("481", nrow(val481_largo))
```

```
sensprototest481 <- ggplot(val481_largo %>%
    drop_na() %>%
    filter(SPR < 0.65), aes(x = SPR, y = Parameter)) + geom_boxplot(aes(fill = ifelse(Parameter ==
    "Linf60", "red", "white")), trim = TRUE, position = position_dodge(0.9),
    adjust = 1/5) + geom_vline(xintercept = 0.75, colour = "#006d2c",
    alpha = 0.5, linetype = 2) + geom_vline(xintercept = 0.2,
    colour = "#bd0026", alpha = 0.5) + geom_jitter(aes(color = ifelse(Parameter ==
    "Linf60", "red", "black")), alpha = 0.4, height = 0, width = 0.1) +
    labs(x = "SPR", y = "VB Parameters tested") + theme_minimal() +
    theme(legend.position = "top", axis.text.x = element_text(angle = 90,
        hjust = 2), panel.background = element_rect(), panel.grid.major = element_blank(),
        panel.grid.minor = element_blank()) + xlim(0, 1) + scale_fill_identity() +
    scale_color_identity()
```

Now, sensitivity analysis to k to 48.1

```
# ciclo de ajuste para 481
for (i in c(2, 0.5, 0.3)) {
    nombre_objeto <- paste0("MyPars_", i)  # Nombre del objeto S4 en cada iteración
    nombre_variable <- paste0("fit481", i)  # Nuevo nombre para el resultado

    # Obtener el objeto S4 correspondiente
    objeto <- get(nombre_objeto)

    # Ejecutar LBSPRfit() y asignar el resultado a una
    # nueva variable con nombre único
```

```
    assign(nombre_variable, LBSPRfit(objeto, Len481), envir = .GlobalEnv)
}
```

```
valprofit481 <- as.data.frame(cbind(fit4810.3@Years, fit4810.3@SPR,
    fit4810.5@SPR, fit4812@SPR))
colnames(valprofit481) <- c("Years", "High", "Med", "Low")

valprofit481_largo <- pivot_longer(valprofit481, cols = c(2:4,
    ), names_to = "Parameter", values_to = "SPR")
valprofit481_largo$SPRstra <- rep("481", nrow(valprofit481_largo))
```

```
senspr481 <- ggplot(valprofit481_largo %>%
    filter(SPR < 0.99) %>%
    drop_na() %>%
    mutate(Parameter = factor(Parameter, levels = c("Low", "Med",
        "High"))), aes(x = Parameter, y = SPR)) + geom_boxplot(aes(fill = ifelse(Parameter ==
    "Med", "red", "white")), color = "black") + geom_hline(yintercept = 0.75,
    colour = "#006d2c", alpha = 0.5, linetype = "dashed") + geom_hline(yintercept = 0.2,
    colour = "#bd0026", alpha = 0.5) + geom_jitter(aes(color = ifelse(Parameter ==
    "Med", "red", "black")), alpha = 0.4, width = 0.1) + labs(y = "SPR",
    x = "Growth Scenario") + theme_minimal() + theme(legend.position = "none",
    panel.background = element_rect(), panel.grid.major = element_blank(),
    panel.grid.minor = element_blank()) + scale_fill_identity() +
    scale_color_identity()
```

Finally, join plot to 48.1 analysis; `spr481plot`, `sensprototest481`, `senspro481` (Figure 10).

```
ggarrange(spr481plot, sensprototest481, senspr481, ncol = 3,
    labels = c("A", "B", "C"))
```

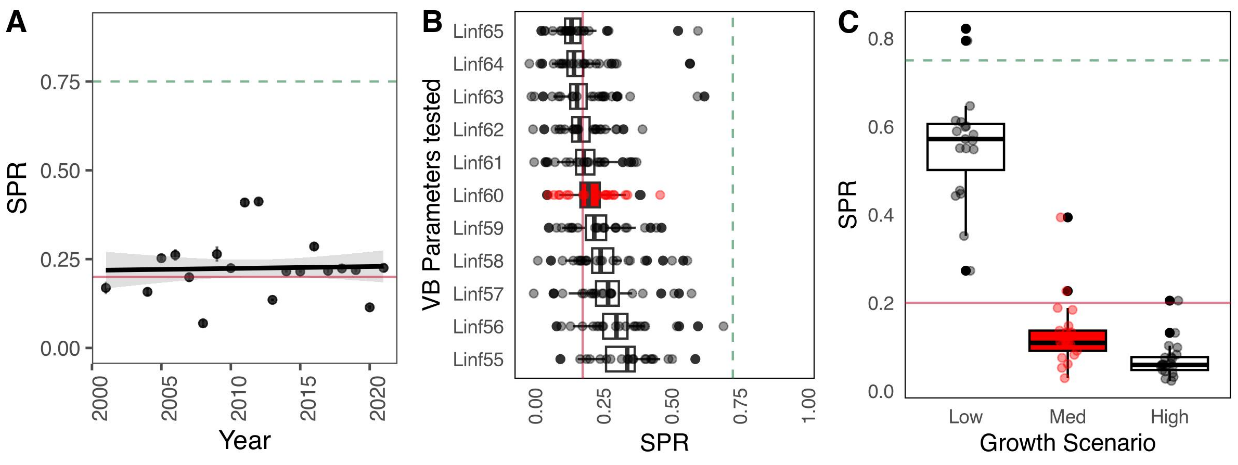

Figure 10: Plot to SPR in different context to 48.1 subarea

GLOSSARY

Table 6: Glossary of Technical Terms

Term	Definition
**Intrinsic Productivity**	Capacity of a population to reproduce and sustain itself, which depends on reproductive and somatic conditions.
**SPR**	Spawning Potential Ratio. Ratio of egg production per recruit under fishing to unfished conditions; key BRP.
**LBSPR**	Length-Based Spawning Potential Ratio model used to estimate stock status from length data.
**VBGF**	Von Bertalanffy Growth Function. Growth model describing length-at-age through parameters Linf, k, and t0.
**Linf**	Asymptotic length; theoretical maximum body size.
**k**	Growth coefficient; rate at which individuals approach Linf.
**M/k**	Life-history invariant representing growth–mortality balance and productivity.
**M**	Natural Mortality. Instantaneous mortality in the absence of fishing.
**F/M**	Relative measure of fishing mortality compared to natural mortality.
**Maturity Ogive**	Logistic function describing proportion of mature individuals by length.
**L50**	Length at which half the population is mature.
**L95**	Length where 95% of individuals are mature.
**CVL**	Coefficient of variation in length used in LBSPR.
**Recruitment**	Entry of new individuals into the population or fishery.
**Recruitment Variability**	Temporal fluctuation in the number of recruits influencing productivity.
**EPR**	Egg Production per Recruit. Lifetime reproductive output of a recruit, fished or unfished.
**Growth Proxy**	Use of length metrics as indicators of physiological conditions.
**Length Composition**	Frequency distribution of observed total lengths from fishery-dependent data.
**Selectivity**	Probability that an individual of a given size enters the fishery.
**SL50**	Lengths at 50% and 95% probability of gear selectivity.
**SL95**	Length at 95% probability of gear selectivity.
**Fishery-Dependent Data**	Data collected onboard vessels such as SISO length compositions.
**Steady-State Distribution**	Expected equilibrium length distribution under constant F and M.
**BRP**	Biological Reference Point. Benchmark to evaluate stock status (e.g., SPR20%, SPR75%).
**LRP**	Limit Reference Point. Threshold below which the stock status is considered impaired (SPR20%).
**TRP**	Target Reference Point. Desired stock status objective (SPR75% for krill).
**MSY**	Maximum Sustainable Yield. Largest long-term average catch that can be sustainably taken.
**Hockey-Stick HCR**	Piecewise-linear harvest control rule used in fisheries management.
**Pseudo-Cohort Analysis**	Length-based analytical approach using multiple length frequencies (LBPA).

**Data-Poor Assessment**	Stock assessment using limited inputs
**Integrated Assessment Model**	Model synthesizing multiple data types within a unified population framework.
**MP**	Management Procedure. Pre-agreed algorithm determining harvest decisions under uncertainty.
**HCR**	Harvest Control Rule. Algorithm linking observed indicators (e.g., SPR) to allowed catch.
**Catch Limit**	The total combined catch of Euphausia superba in Statistical Subareas 48.1, 48.2, 48.3 and 48.4 shall be limited to 5.61 million tonnes in any fishing season (CM 51-07).
**Trigger Level**	Total combined catch in Statistical Subareas 48.1, 48.2, 48.3 and 48.4 shall be further limited to 620 000 tonnes in any fishing season (CM 51-07).
**CCAMLR**	Commission for the Conservation of Antarctic Marine Living Resources.
**SISO**	CCAMLR's Scheme of International Scientific Observation providing fishery monitoring data.
**Subarea 48.1**	Main krill fishing area along the Western Antarctic Peninsula.
**Management Strata**	Spatial fishery management units in 48.1 (BS, EI, GS, JOIN, SSWI).
**SSMU**	Small-Scale Management Unit used for ecosystem-based krill management.
**CM 51-07**	Conservation Measure specifying spatial distribution of the trigger level; expired in 2024.
**Spatial Heterogeneity**	Differences in productivity or population structure across regions.
**WAP**	Western Antarctic Peninsula, a region experiencing rapid warming and sea-ice decline.
**SST**	Sea Surface Temperature; key environmental driver affecting krill growth and productivity.
**SIC**	Sea Ice Concentration derived from Nimbus-7; influences recruitment.
**Chlorophyll-a**	Proxy for primary productivity and food availability.
**Environmental Covariate**	External variable influencing krill growth dynamics.
**GLMM**	Mixed-effects model incorporating fixed effects and random structure.
**Pearson Correlation**	Correlation metric assessing relationship between length and environmental variables.
**Rasterization**	Conversion of spatial satellite data to uniform grids.
**sf**	R package for handling spatial vector data.
**ncdf4**	R interface for working with NetCDF satellite and oceanographic data.

CODE REPOSITORY

The data, codes and another documents of this exercise can be found in the following link LBSPR-Krill

REFERENCES

Goodyear, C. P. (1993). Spawning stock biomass per recruit in fisheries management: foundation and current use. . *Risk Evaluation and Biological Reference Points for Fisheries Management*, *120*, 67–81.

Hordyk, A. R., Ono, K., Prince, J. D., & Walters, C. J. (2016). A simple length-structured model based on life history ratios and incorporating size-dependent selectivity: application to spawning potential ratios for data-poor stocks. *Canadian Journal of Fisheries and Aquatic Sciences*, *73*(12), 1787–1799. https://doi.org/10.1139/cjfas-2015-0422

Hordyk, A., Ono, K., Sainsbury, K., Loneragan, N., & Prince, J. (2014). Some explorations of the life history ratios to describe length composition, spawning-per-recruit, and the spawning potential ratio. *ICES Journal of Marine Science*, *72*(1), 204–216. https://doi.org/10.1093/icesjms/fst235

Maschette, D., Wotherspoon, S., Kawaguchi, S., & Ziegler, P. (2021). *WG-FSA-2021/39 29. Grym assessment for Subarea 48.1 Euphausia superba populations*. CCAMLR.

Thanassekos, S., Cox, M. J., & Reid, K. (2014). Investigating the effect of recruitment variability on length-based recruitment indices for antarctic krill using an individual-based population dynamics model. *PLoS ONE*, *9*(12), 1–20. https://doi.org/10.1371/journal.pone.0114378

Zhou, S., Yin, S., Thorson, J. T., Smith, A. D. M., Fuller, M., & Walters, C. J. (2012). Linking fishing mortality reference points to life history traits: an empirical study. *Canadian Journal of Fisheries and Aquatic Sciences*, *69*(8), 1292–1301. https://doi.org/10.1139/f2012-060
