# Supplementary material for: Disparate estimates of intrinsic productivity for Antarctic krill across small spatial scales under a rapidly changing ocean
Source: PLoS One. 2026 Jan 30;21(1):e0322671. doi: 10.1371/journal.pone.0322671 (PMC12857959; doi:10.1371/journal.pone.0322671)
Supplement: S2 File — (PDF) [file pone.0322671.s002.pdf]

Supporting Information 2

Code and analysis for identifying environmental influences on krill length using correlation and mixed-effects models across spatial and temporal scales

02 June, 2025

Contents

Background **2**

Methodology **3**

- Spatial heterogeneity . . . 3
- Length composition data as indicator of recruitment . . . 4
- Test Correlation . . . 6
- Variable Distribution . . . 8
- Models . . . 9

Results **11**

Conclusion **14**

Code Repository **15**

References **16**

Background

This study aims to explore the relationship between environmental variables and the population dynamics of krill (*Euphausia superba*), incorporating spatial complexity and biological components such as length data from fishery monitoring. By using lineal models, we assess how environmental factors influence krill recruit across different strata, considering both spatial and temporal scales. By identifying correlations between environmental variables and population or fishery indicators, this study also aims to establish a time series of key environmental factors for potential integration into stock assessment models. This approach follows similar methodologies to Wang et al. (2021), but applied to a longer fishery time series. Our main research question concerns the effects of distinct physical and oceanographic factors in the Southern Ocean on krill population structure. Specifically, we examine whether environmental variability drives changes in krill recruit within the fishery, influencing population dynamics across spatial and temporal dimensions.

Methodology

Spatial heterogeneity

Figure 1 illustrates the spatial heterogeneity of key environmental and population variables across different management units (BS, EI, GS, JOIN, SSWI) in the Antarctic Peninsula, where krill populations are distributed. Biomass and catch trends show substantial variability between strata, with BS and GS exhibiting the highest biomass estimates, whereas JOIN and SSWI display comparatively lower values. Catch levels also differ significantly, with BS and GS experiencing the most intensive exploitation, while SSWI and JOIN have minimal catch records. Environmental variables further highlight this heterogeneity. Sea surface temperature (SST) trends vary among strata, with GS and JOIN exhibiting a slight warming trend over time, while BS and EI remain relatively stable. Sea ice cover differs substantially, with GS showing consistently high coverage, whereas JOIN presents greater fluctuations. Chlorophyll-a (Chl-a) levels, a proxy for primary productivity, also vary across regions, with BS and GS showing declining trends, while EI and SSWI remain relatively stable at lower concentrations. Given these spatial differences in both krill population metrics and environmental conditions, it is essential to analyze and estimate SPR at this local scale. The observed heterogeneity supports the need for spatially explicit management, as krill population dynamics are likely influenced by regional environmental drivers. By incorporating SPR analysis at this resolution, we can provide a spatial explicit framework for sustainable krill management, ensuring that conservation efforts align with local population and ecosystem characteristics.

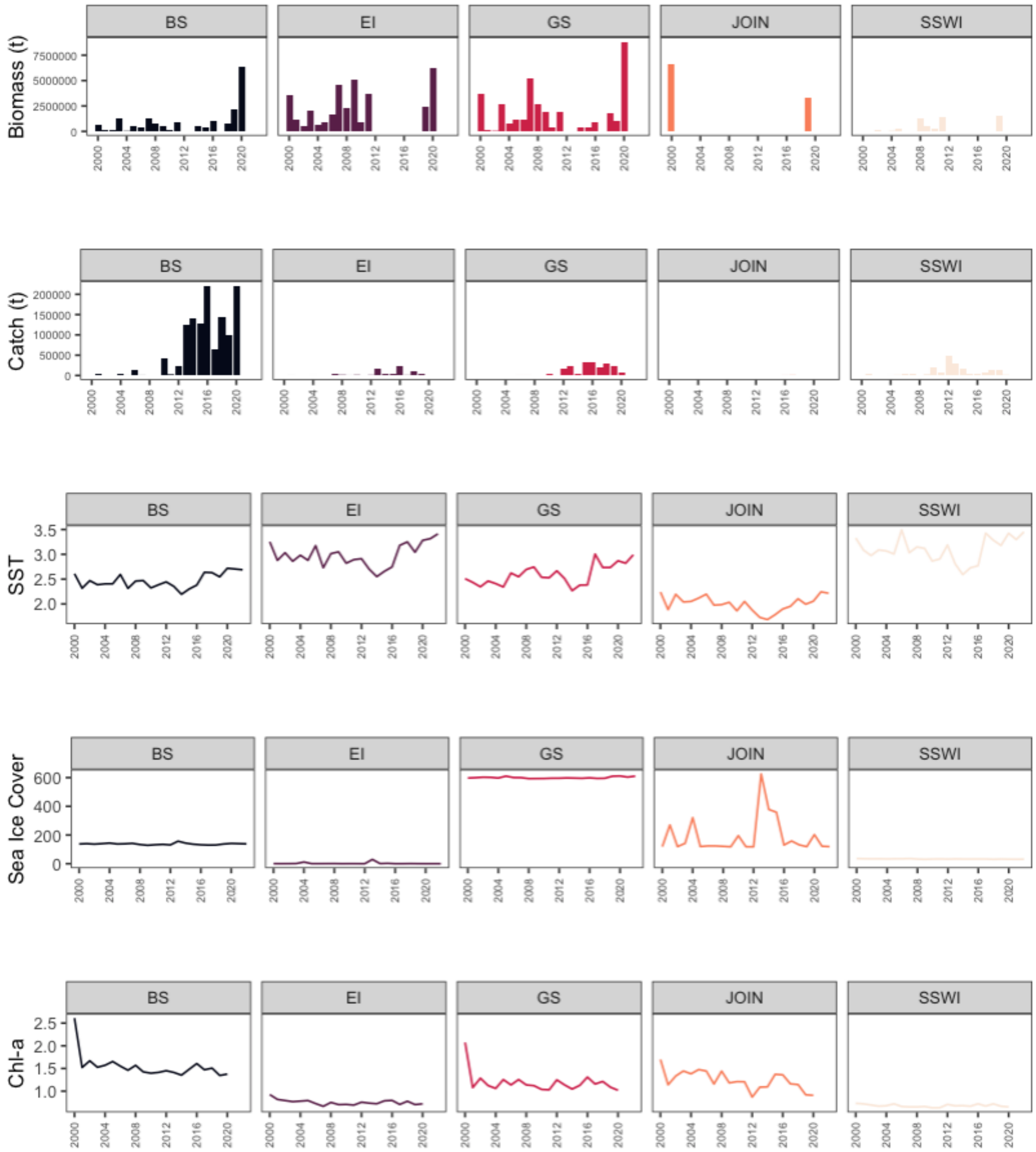

Figure 1: Spatial heterogeneity of krill biomass, catch, and environmental variables across management units in the Antarctic Peninsula (BS, EI, GS, JOIN, SSWI)

Length composition data as indicator of recruitment

This analysis was made follow Perry (2020) method.

The recruitment index is calculated by first determining the proportion of juvenile krill individuals based on their total length, specifically those under a threshold of 3.6 cm. This proportion is calculated as the ratio of juvenile individuals to the total number of krill sampled within a given year and for each identified group (ID). The resulting proportion is then transformed using a logarithmic function of length, which represents the log-transformed recruitment index.

Next, the *PROPLOG* values are standardized to fall within a specific range, typically between -1 and 1. This standardization process is achieved by applying the following equation:

$$\text{PROPLOG2} = \frac{\text{PROPLOG} - \min(\text{PROPLOG})}{\max(\text{PROPLOG}) - \min(\text{PROPLOG})} \times (b - a) + a$$

In this equation:

- **PROPLOG** is the logarithm of the proportion of juvenile krill individuals.
- **PROPLOG2** is the standardized recruitment index, scaled between -1 and 1.
- **min()** and **max()** refer to the minimum and maximum values of **PROPLOG** across all observations.
- **a** and **b** are the lower and upper bounds of the desired range for standardization, which are set to -1 and 1, respectively.

Finally, the standardized index is categorized into two groups based on its value: "positive" for values greater than or equal to 0, and "negative" for values below 0. This categorization allows for further analysis of recruitment trends and patterns within the krill population over time.

This methodology ensures that the recruitment index is both consistent and comparable across different years and groups.

```r
indice_reclutamiento <- sf5 %>%
    filter(length_total_cm < 3.6) %>%
    group_by(Year, ID) %>%
    summarise(PROP = n()/nrow(sf5), .groups = "drop") %>%
    mutate(PROPLOG = log(PROP))

# Paso 2: Estandarizar el índice logarítmico entre -1
# y 1
a <- -1
b <- 1
min_x <- min(indice_reclutamiento$PROPLOG, na.rm = TRUE)
max_x <- max(indice_reclutamiento$PROPLOG, na.rm = TRUE)

indice_reclutamiento <- indice_reclutamiento %>%
    mutate(PROPLOG2 = ((PROPLOG - min_x)/(max_x - min_x)) *
        (b - a) + a)

indice_reclutamiento <- indice_reclutamiento %>%
    mutate(color_index = ifelse(PROPLOG2 >= 0, "positive",
        "negative"))
```

Figure 2 show recruit index by strata.

```r
ggplot(indice_reclutamiento %>%
    filter(ID != "Outer"), aes(x = Year, y = PROPLOG2, fill = color_index)) +
    geom_col(position = "dodge") + facet_wrap(~ID, ncol = 5) +
    scale_fill_manual(values = c(positive = "red", negative = "black")) +
    labs(title = "", y = "Recruit Index", x = "", fill = "") +
    theme_bw() + theme(axis.text.x = element_text(angle = 90,
    hjust = 1), axis.text = element_text(size = 7), legend.position = "none")
```

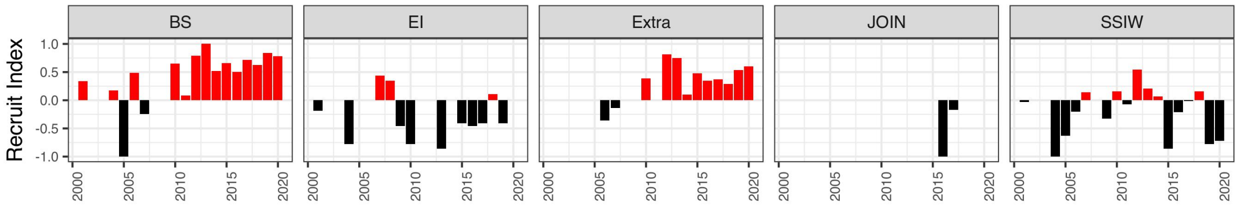

Figure 2: Krill Recruit index by strata

```
data_large2 <- data_large2 %>%
    rename(Year = ANO)

# Paso 2: Unir por ID y Year
data_completa <- left_join(data_large2, indice_reclutamiento,
    by = c("ID", "Year"))
# saveRDS(data_completa,
# 'data/Data_recruit_env.Rdata')
```

Test Correlation

The Pearson test are statistical methods used to assess the correlation between two variables. The Pearson test evaluates the linear correlation between two continuous variables. In the case of the Pearson test, the degree of association between two continuous variables is measured through a correlation coefficient that varies between -1 and 1. A value of 1 indicates a perfectly positive correlation, a value of -1 indicates a perfectly negative correlation, and a value of 0 indicates no correlation between the two variables (McCulloch & Searle, 2001).

- Pearson Product-Moment Coefficient

This is the most widely used correlation analysis formula, which measures the strength of the 'linear' relationships between the raw data from both variables, rather than their ranks. This is an dimensionless coefficient, meaning that there are no data-related boundaries to be considered when conducting analyses with this formula, which is a reason why this coefficient is the first formula researchers try.

$$r = 1 - \frac{6\sum_{i=1}^{n} D_i^n}{n(n^2-1)}$$

```
data_corr <- data_completa %>%
    dplyr::select(-ID, -color_index) %>%
    dplyr::mutate(across(where(is.character), as.numeric)) %>%
    cor(method = "pearson", use = "complete.obs")
```

```
ggcorrplot(data_corr, method = "circle", type = "lower",
    colors = c("green", "white", "yellow"), lab = TRUE,
    outline.col = "white", ggtheme = theme_minimal())
```

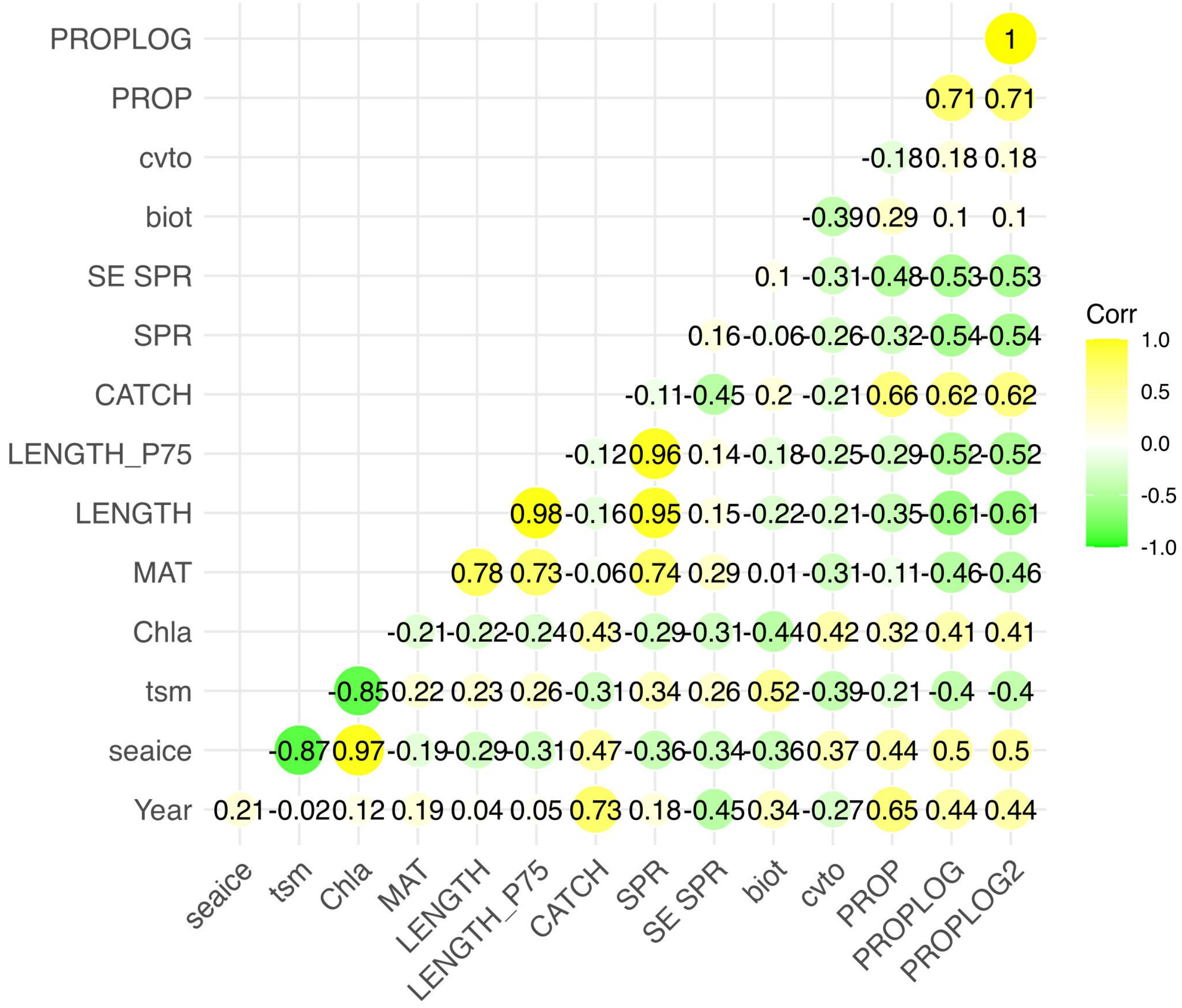

Figure 3: Coorrelation plot to different variables.

The correlation matrix provides valuable insights into the relationships among the variables (Figure 3):

This plot is a correlation matrix that visualizes the pairwise relationships between variables using both color intensity and numerical values. Yellow indicates strong positive correlations (close to +1), green indicates strong negative correlations (close to −1), and lighter colors represent weaker correlations (closer to 0). The size of each circle is also proportional to the strength of the correlation.

The variables PROLOG and PROP are highly positively correlated, with values around 0.70–0.71. Similarly, MAT (presumably maturity) shows strong positive correlations with LENGTH (0.78) and LENGTH_P75 (0.76), suggesting that as individuals grow larger, their maturity increases.

On the other hand, seaice is negatively correlated with several biological variables: it has a correlation of –0.97 with tsm, –0.79 with Chla, –0.78 with MAT, and –0.81 with LENGTH. This suggests that higher sea ice coverage is associated with lower temperature, lower chlorophyll a concentration, lower maturity, and smaller lengths.

Moderate positive correlations are also observed among SPR, SE SPR, and CATCH, as well as between biot, cvto, and the productivity indices. This pattern may indicate a potential linkage between biological productivity and reproductive metrics.

Variable Distribution

To explore the distribution of numerical variables in our dataset, we conducted a histogram analysis (Figure 4. First, we excluded non-numeric variables (ID, Year, SE SPR, and cvto) to focus on the remaining quantitative data. The results from this exploratory analysis provided essential insights into the characteristics of our dataset, helping to guide subsequent modeling and statistical analyses.

```
data_filtered2 <- data_large2 %>%
    dplyr::select(-ID, -Year, -`SE SPR`, -cvto) %>%
    pivot_longer(cols = everything(), names_to = "Variable",
        values_to = "Valor")

ggplot(data_filtered2, aes(x = Valor)) + geom_histogram(bins = 30,
    fill = "skyblue", color = "black", alpha = 0.7) + facet_wrap(~Variable,
    scales = "free") + theme_minimal() + labs(title = "Variable distribution",
    x = "", y = "Frecuency")
```

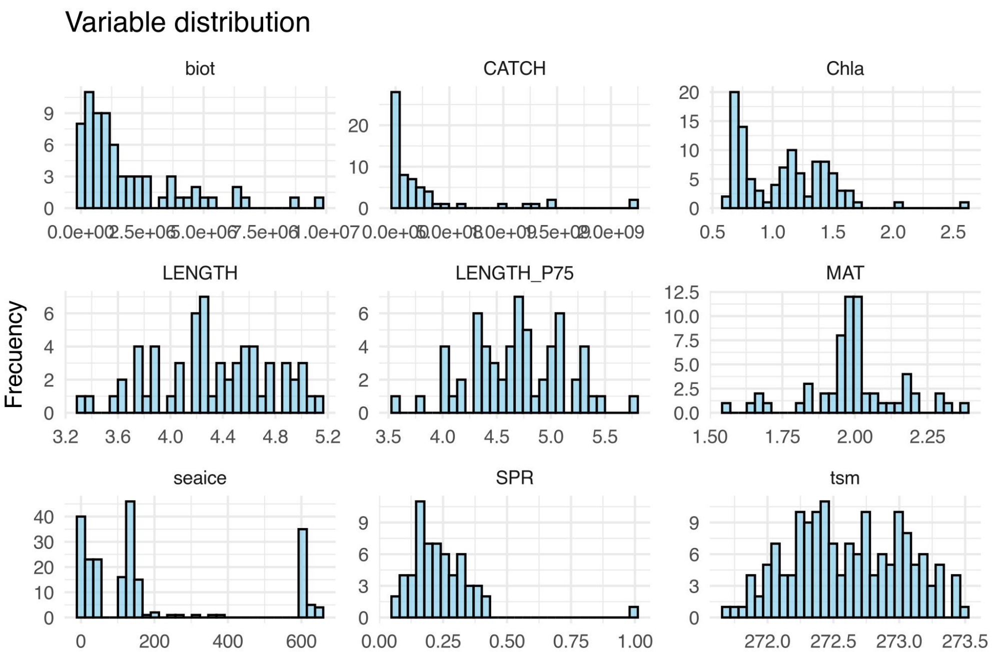

Figure 4: Distribution of numerical variables to test assumtion of regresion models

Models

The analysis of the correlation between the recruitment index (PROPLOG2) and environmental variables will be approached through a series of regression models designed to examine the impact of various environmental factors on the recruitment process of krill. These models progressively include more explanatory variables, allowing us to evaluate the relative contribution of each environmental factor, as well as potential interactions between them, on the recruitment index. Each model represents a different level of complexity and addresses distinct aspects of the potential relationship between recruitment and environmental conditions.

In the first model (Mod 1), the recruitment index is modeled solely as a function of the ID, which represents a baseline level of recruitment. This model serves as a starting point for understanding the overall distribution of recruitment across different IDs without considering any environmental factors.

The second model (Mod 2) introduces *Chla* (chlorophyll a concentration) as an additional explanatory variable. This model will allow for the evaluation of how changes in primary production, represented by chlorophyll a, may influence recruitment. The inclusion of chlorophyll a will help determine whether biological productivity, as reflected by phytoplankton concentrations, plays a role in shaping recruitment dynamics.

The third model (Mod 3) further expands on the previous one by including *TSM* (total suspended matter), which is a measure of the particulate matter in the water column. This model investigates whether changes in suspended matter, which could affect light penetration and, consequently, primary production, influence recruitment. The model will examine how both chlorophyll a and TSM, as separate environmental variables, contribute to the variation in recruitment.

The fourth model (Mod 4) incorporates *seaice* as an additional explanatory factor. Sea ice presence and its seasonal variations have been shown to impact the distribution and availability of krill habitats. By including sea ice in the model, we aim to explore the potential impact of this variable on recruitment, alongside chlorophyll a and suspended matter.

The fifth model (Mod 5) introduces an interaction term between *TSM* and *Chla*, which allows for the examination of how the combined effect of these two environmental factors might influence recruitment. This interaction term will help determine whether the relationship between TSM and recruitment is modified by the presence of chlorophyll a, or vice versa. This model will help us understand whether the effects of primary production and suspended matter on recruitment are synergistic or independent.

Finally, the model labeled "Mod 5 Lag" considers a lag effect for both TSM and Chla, where the values of these variables from the previous time point are included. This lag effect will help us assess whether past environmental conditions influence current recruitment levels, reflecting the potential delayed response of the ecosystem to changes in these variables.

By comparing the results of these models, we can systematically evaluate the contribution of each environmental factor and their interactions to the recruitment index. The inclusion of lags in the final model will provide insights into the temporal dynamics of the recruitment process, shedding light on how past environmental conditions might influence current recruitment patterns. This methodology allows for a comprehensive assessment of the factors affecting krill recruitment and their potential implications for the population dynamics of this important marine species.

$$\text{Mod 1:} \quad PROPLOG2_i = \beta_0 + \beta_1 ID_i + \epsilon_i$$

$$\text{Mod 2:} \quad PROPLOG2_i = \beta_0 + \beta_1 ID_i + \beta_2 Chla_i + \epsilon_i$$

$$\text{Mod 3:} \quad PROPLOG2_i = \beta_0 + \beta_1 ID_i + \beta_2 Chla_i + \beta_3 TSM_i + \epsilon_i$$

$$\text{Mod 4:} \quad PROPLOG2_i = \beta_0 + \beta_1 ID_i + \beta_2 Chla_i + \beta_3 TSM_i + \beta_4 seaice_i + \epsilon_i$$

$$\text{Mod 5:} \quad PROPLOG2_i = \beta_0 + \beta_1 ID_i + \beta_2 seaice_i + \beta_3 TSM_i + \beta_4 Chla_i + \beta_5 (TSM \times Chla)_i + \epsilon_i$$

$$\text{Mod 5 Lag:} \quad PROPLOG2_i = \beta_0 + \beta_1 ID_i + \beta_2 seaice_i + \beta_3 TSM_{i-1} + \beta_4 Chla_{i-1} + \beta_5 (TSM_{i-1} \times Chla_{i-1})_i + \epsilon_i$$

This represents each model in mathematical terms, where β are the coefficients and ϵ_i is the error term.

```r
# Prepara los datos Base filtrada y normalizada
data_modelo <- data_completa %>%
    drop_na(PROPLOG2, Chla, tsm, seaice) %>%
    filter(Year > 1999) %>%
    mutate(tsm = scale(tsm), Chla = scale(Chla), seaice = scale(seaice))

# Modelo 1: Solo ID
mod1_R <- glm(PROPLOG2 ~ ID + Year, data = data_modelo)

# Modelo 2: ID + Chla
mod2_R <- glm(PROPLOG2 ~ ID + Year + Chla, data = data_modelo)

# Modelo 3: ID + Chla + tsm
mod3_R <- glm(PROPLOG2 ~ ID + Year + Chla + tsm, data = data_modelo)

# Modelo 4: ID + Chla + tsm + seaice
mod4_R <- glm(PROPLOG2 ~ ID + Year + Chla + tsm + seaice,
    data = data_modelo)

# Modelo 5: ID + seaice + interacción tsm * Chla
mod5_R <- glm(PROPLOG2 ~ ID + Year + seaice + tsm * Chla,
    data = data_modelo)

# Crear variables con retardo temporal (lag 1 año)
data_lag <- data_modelo %>%
    arrange(ID, Year) %>%
    group_by(ID) %>%
    mutate(tsm_lag = lag(tsm, 1), Chla_lag = lag(Chla, 1),
        seaice_lag = lag(seaice, 1)) %>%
    ungroup() %>%
    drop_na(tsm_lag, Chla_lag, seaice_lag)

# Modelo 5 con retardo
mod5_R_lag <- glm(PROPLOG2 ~ ID + Year + seaice_lag + tsm_lag *
    Chla_lag, data = data_lag)
```

Table 1: Model Performance Comparison

Name	Model	R2	RMSE	Sigma	AIC_wt	AICc_wt	BIC_wt	Performance_Score
mod2_R	glm	0.594	0.383	0.419	0.405	0.600	0.574	0.891
mod3_R	glm	0.610	0.375	0.419	0.279	0.201	0.196	0.659
mod5_R	glm	0.635	0.363	0.423	0.102	0.010	0.018	0.533
mod4_R	glm	0.619	0.370	0.423	0.145	0.043	0.051	0.529
mod1_R	glm	0.504	0.423	0.454	0.055	0.145	0.157	0.180
mod5_R_lag	glm	0.528	0.407	0.486	0.015	0.001	0.004	0.074

Results

```r
model_comparison <- compare_performance(mod1_R, mod2_R,
    mod3_R, mod4_R, mod5_R, mod5_R_lag, rank = TRUE, verbose = FALSE)

model_comparison %>%
    kable(format = "latex", digits = 3, align = "c", caption = "Model Performance Comparison") %>%
    kable_styling(full_width = TRUE, bootstrap_options = c("striped",
        "hover", "condensed", "responsive")) %>%
    scroll_box(width = "100%", height = "400px")
```

The Figure 5 show graphically the performance in the models.

```r
plot(compare_performance(mod1_R, mod2_R, mod3_R, mod4_R,
    mod5_R, mod5_R_lag, rank = TRUE, verbose = FALSE))
```

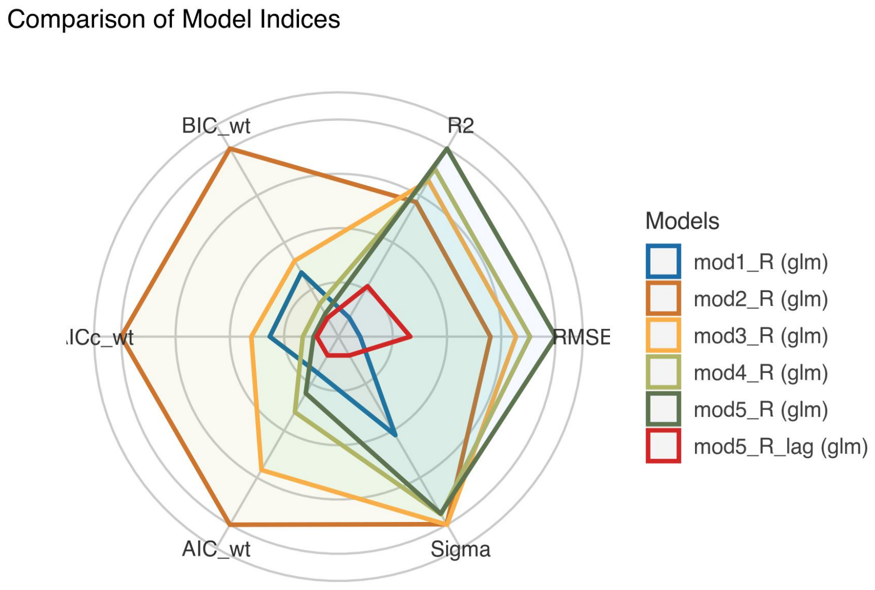

Figure 5: Comparision the performance and quality of several models

In our *best model* `mod2_R` we fitted a linear mixed model (estimated using ML and nloptwrap optimizer) to predict `Recruit` with `ID`, `seaice`, `tsm` and `Chla`.

Table 2: Summary of GLM model for PROPLOG2

Term	Estimate	Std. Error	t value	Pr(>|t|)
(Intercept)	-26.0863	30.8799	-0.8448	0.4063
IDEI	-2.6355	0.8193	-3.2167	0.0036
IDJOIN	-1.6326	0.3821	-4.2721	0.0002
Year	0.0136	0.0153	0.8881	0.3830
Chla	-0.9401	0.3997	-2.3521	0.0268

```r
tabla_coef <- broom::tidy(mod2_R)

# Redondear columnas numéricas
tabla_coef <- tabla_coef %>%
    mutate(across(where(is.numeric), round, 4))

# Crear tabla HTML
tabla_coef %>%
    kable(format = "latex", escape = FALSE, caption = "Summary of GLM model for PROPLOG2",
        col.names = c("Term", "Estimate", "Std. Error",
            "t value", "Pr(>|t|)")) %>%
    kable_styling(full_width = FALSE, bootstrap_options = c("striped",
        "hover", "condensed"))
```

The results from the generalized linear model (GLM) with the formula `PROPLOG2 ~ ID + Year + Chla` are as follows:

The coefficients are as follows:

- **(Intercept)**: -26.08628, p = 0.406254
  The intercept is negative (-26.08628) but not statistically significant (p = 0.406), indicating that it does not have a meaningful effect on the response variable.
- **IDEI (ID)**: -2.63547, p = 0.003567
  The coefficient for `IDEI` is negative (-2.63547) and statistically significant (p = 0.003567), suggesting that an increase in `IDEI` leads to a decrease in `PROPLOG2`.
- **IDJOIN (ID)**: -1.63256, p = 0.000246
  The coefficient for `IDJOIN` is also negative (-1.63256) and highly statistically significant (p = 0.000246). This indicates that an increase in `IDJOIN` is associated with a decrease in `PROPLOG2`.
- **Year (Año)**: 0.01358, p = 0.382954
  The coefficient for `Year` is positive (0.01358), but it is not statistically significant (p = 0.383), suggesting that `Year` does not have a meaningful effect on `PROPLOG2`.
- **Chla (Chlorophyll a)**: -0.94014, p = 0.026849
  The coefficient for `Chla` is negative (-0.94014) and statistically significant (p = 0.026849), indicating that an increase in `Chla` leads to a decrease in `PROPLOG2`.

Regarding the model fit:

- The **null deviance** is 10.8104, which represents the deviance of a model with no predictors.
- The **residual deviance** is 4.3936, which is the deviance of the fitted model.
- The **AIC (Akaike Information Criterion)** is 39.505, which helps to compare this model to others (lower values indicate a better fit).

The model uses two iterations of Fisher scoring for optimization.

In summary, the model suggests that `IDEI`, `IDJOIN`, and `Chla` have significant effects on `PROPLOG2`, while `Year` does not. The residual deviance and AIC indicate a reasonable fit, but the model could potentially be improved by including more predictors or transformations.

```
data_large3 <- data_completa %>%
    arrange(ID, Year) %>%
    group_by(ID) %>%
    mutate(SPR_lag = lag(SPR)) %>%
    ungroup()
```

Figure 6 show negative relation between recruit index and Chl-a.

```
ggplot(data_large3 %>%
    filter(Year > 2000, ID == "BS"), aes(x = Chla, y = PROPLOG2)) +
    geom_point(size = 3, alpha = 0.7, aes(color = as.factor(Year))) +
    geom_smooth(method = "lm", se = TRUE, color = "black") +
    stat_cor(method = "pearson", label.y.npc = "bottom") +
    # facet_wrap(~ID, scales = 'free', ncol=5) +
theme_minimal() + scale_color_viridis_d(option = "F", name = "") +
    labs(title = "", x = "Chl-a", y = "Recruit Index", color = "Año")
```

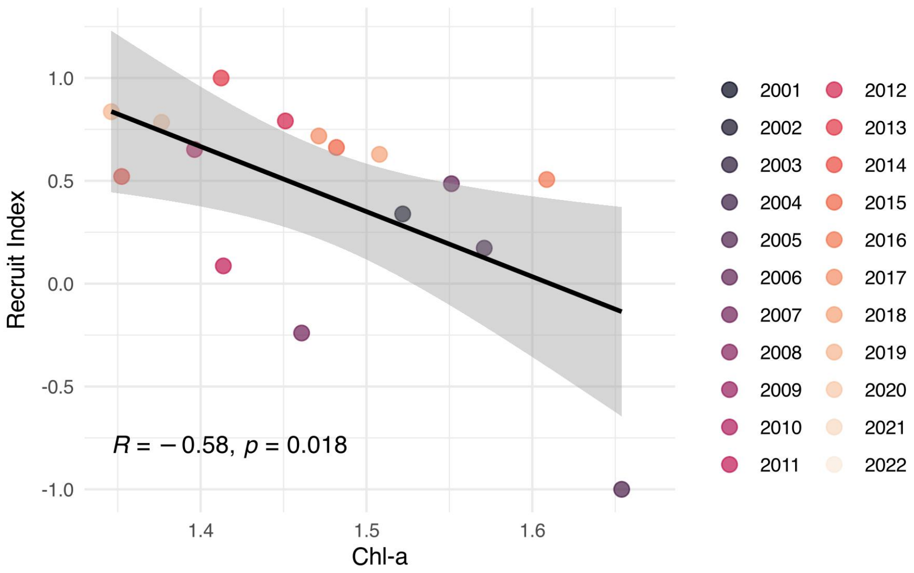

Figure 6: Relationship between mean annual chlorophyll-a concentration (Chl-a) and the standardized krill recruit index across years (2001-2022). Each point represents a year and is color-coded accordingly. The black line represents a linear regression fit with a 95% confidence interval (shaded area). A significant negative correlation was found between Chl-a and the recruit index (*R* = - 0.58, *p* = 0.018)

Conclusion

The results of Model 2, which includes `ID`, `Year`, and `Chla` as predictors for `PROPLOG2`, reveal several significant findings. The negative coefficient for `IDEI` indicates that as `IDEI` increases, the response variable `PROPLOG2` decreases, with a statistically significant p-value (0.003567). Similarly, the negative coefficient for `IDJOIN` suggests that an increase in `IDJOIN` also leads to a decrease in `PROPLOG2`, and this relationship is highly significant ($p = 0.000246$).

While the `Year` variable shows a positive but non-significant effect ($p = 0.382954$), suggesting that the year does not have a notable impact on `PROPLOG2`, the `Chla` variable exhibits a significant negative relationship with the response variable ($p = 0.026849$). This suggests that higher levels of chlorophyll-a (`Chla`) are associated with lower values of `PROPLOG2`.

The model's AIC value (39.505) and residual deviance (4.3936) suggest a reasonable fit, with the predictors included contributing to explaining the variability in the response variable. However, given that `Year` is not statistically significant, further investigation could explore alternative variables or transformations to improve the model's explanatory power.

Our analysis aimed to explore the spatial variability in krill *LENGTH* and the 75th percentile of length (*LENGTH_P75*) while incorporating key environmental covariates, such as chlorophyll-a concentration (Chla), sea surface temperature (TSM), and sea ice cover. Using mixed-effects models, we evaluated the influence of these environmental factors on krill growth across different spatial strata (ID) while accounting for temporal variability by including year (Year) as a random effect. The inclusion of interactions between TSM and Chla allowed us to capture potential synergistic or antagonistic effects of these variables, providing insights into how environmental variability drives size distribution across regions.

Among the tested models, **Model 5** which includes spatial strata (ID), sea ice, and the interaction between TSM and Chla—performed best based on AIC selection criteria. The model revealed significant annual and spatial variability in *LENGTH*, suggesting that both temporal and spatial factors play a crucial role in shaping this response variable. Among the environmental predictors, Chla exhibited a notable effect, indicating its potential influence on LENGTH, while the effects of tsm and seaice were more uncertain, with wide confidence intervals suggesting a higher degree of variability or weaker associations. Additionally, the random effects associated with ID were significant, highlighting differences among stratas that may be driven by intrinsic biological factors. These findings emphasize the importance of accounting for both fixed and random effects when analyzing variations in LENGTH over time and across individuals.

On one hand, the mixed-effects models confirm the influence of spatial structure, where each stratum represents a distinct environmental context affecting both the dependent variable (krill sizes) and independent variables (environmental factors). This highlights the importance of considering spatial heterogeneity in growth studies.

Additionally, our results corroborate the influence of environmental variables on krill size variability. Among them, chlorophyll-a emerged as the most significant factor, with a negative effect on krill size. This suggests that higher chlorophyll concentrations lead to increased recruitment, as the greater availability of phytoplankton provides a more abundant substrate for the krill population, thereby shifting the size structure toward smaller individuals.

Overall, these findings demonstrate that krill population structure is shaped not only by fishing pressure but also by environmental conditions. By incorporating these environmental components into stock assessment models, we enhance our understanding of krill population dynamics in the Antarctic Peninsula, particularly in Subarea 48.1, and improve the ecological realism of predictive models such as LBSPR.

Code Repository

The data, codes and other documents of this analysis can be found in the following link Environment and Krill Recruit relation

References

McCulloch, C. E., & Searle, S. R. (2001). *Generalized, Linear, and Mixed Models* (Vol. 45, pp. 99–99). https://doi.org/10.1198/tech.2003.s13

Perry, F. (2020). *Antarctic krill recruitment in the south-west Atlantic sector of the Southern Ocean* (p. 310) [PhD thesis]. UNIVERSITY OF SOUTHAMPTON.

Wang, R., Song, P., Li, Y., & Lin, L. (2021). An Integrated, Size-Structured Stock Assessment of Antarctic Krill, Euphausia superba. *Frontiers in Marine Science*, *8*(November), 1–15. https://doi.org/10.3389/fmars.2021.710544
