# Supplementary material for: Disparate estimates of intrinsic productivity for Antarctic krill across small spatial scales under a rapidly changing ocean
Source: PLoS One. 2026 Jan 30;21(1):e0322671. doi: 10.1371/journal.pone.0322671 (PMC12857959; doi:10.1371/journal.pone.0322671)
Supplement: Attachment — Submitted filename: Response to Reviewers.docx [file pone.0322671.s004.docx]

**Editor Observations and Responses**

Obs 1: When submitting your revision, we need you to address these additional requirements.

1. Please ensure that your manuscript meets PLOS ONE's style requirements, including those for file naming. The PLOS ONE style templates can be found at

<https://journals.plos.org/plosone/s/file?id=wjVg/PLOSOne_formatting_sample_main_body.pdf> and

<https://journals.plos.org/plosone/s/file?id=ba62/PLOSOne_formatting_sample_title_authors_affiliations.pdf>

Reply: Fixed Author and Affiliation issues in “Revised Manuscript with Track Changes” and “Manuscript” regarding guidelines

Obs 2: Thank you for stating the following financial disclosure: [CC; FS: INACH “Marine Protected Areas” Program (Grant No. 2409052). CC: ANID/Millennium Science Initiative Program (Grant No. ICN2021_002). MM: Doctorate Scholarship from ANID/Centro IDEAL, FONDAP at the Universidad Austral de Chile. (Grant No. 15150003) MM: Commission for the Conservation of Antarctic Marine Living Resources (CCAMLR) Scholarship Scheme (2023-2024)].

Please state what role the funders took in the study.  If the funders had no role, please state: ""The funders had no role in study design, data collection and analysis, decision to publish, or preparation of the manuscript.""

If this statement is not correct you must amend it as needed.

Please include this amended Role of Funder statement in your cover letter; we will change the online submission form on your behalf.

Reply: We appreciate the observation regarding the role of the funders. We confirm that we will include the following statement in our revised cover letter:

*"The funders had no role in study design, data collection and analysis, decision to publish, or preparation of the manuscript."*

Please proceed with updating the online submission form accordingly.

Obs 3: Thank you for stating the following in the Acknowledgments Section of your manuscript: [This research was supported by the following funding sources: the INACH “Marine Protected Areas” Program (Grant No. 2409052), the ANID/Millennium Science Initiative Program (Grant No. ICN2021_002), the CCAMLR Scholarship Scheme (2023-2024), and the Doctorate Scholarship from CENTRO-IDEAL at the Universidad Austral de Chile. The authors extend their gratitude to the Secretariat of the Commission for the Conservation of Antarctic Marine Living Resources (CCAMLR) for providing access to the krill fishery data, which was instrumental to this study.]

We note that you have provided funding information that is not currently declared in your Funding Statement. However, funding information should not appear in the Acknowledgments section or other areas of your manuscript. We will only publish funding information present in the Funding Statement section of the online submission form.

Please remove any funding-related text from the manuscript and let us know how you would like to update your Funding Statement. Currently, your Funding Statement reads as follows: [CC; FS: INACH “Marine Protected Areas” Program (Grant No. 2409052). CC: ANID/Millennium Science Initiative Program (Grant No. ICN2021_002). MM: Doctorate Scholarship from ANID/Centro IDEAL, FONDAP at the Universidad Austral de Chile. (Grant No. 15150003) MM: Commission for the Conservation of Antarctic Marine Living Resources (CCAMLR) Scholarship Scheme (2023-2024)].

Please include your amended statements within your cover letter; we will change the online submission form on your behalf.

Reply: We acknowledge the observation and confirm that we have removed all funding-related information from the Acknowledgments section of the new manuscript, in accordance with the editor's recommendation. Only the expressions of gratitude remain, as suggested.

Additionally, we have incorporated the complete funding information in the revised cover letter, as requested. Please proceed with updating the Funding Statement section of the online submission form accordingly.

Obs 4: In the online submission form, you indicated that [The original data used in this study are available through the Commission for the Conservation of Antarctic Marine Living Resources (CCAMLR) and can be obtained upon request by contacting the CCAMLR Data Centre.].

All PLOS journals now require all data underlying the findings described in their manuscript to be freely available to other researchers, either 1. In a public repository, 2. Within the manuscript itself, or 3. Uploaded as supplementary information.

This policy applies to all data except where public deposition would breach compliance with the protocol approved by your research ethics board. If your data cannot be made publicly available for ethical or legal reasons (e.g., public availability would compromise patient privacy), please explain your reasons on resubmission and your exemption request will be escalated for approval.

Reply: We appreciate the opportunity to clarify our Data Availability statement.

The original data used in this study are managed by the Commission for the Conservation of Antarctic Marine Living Resources (CCAMLR), which applies strict policies for sharing data generated within the jurisdiction of its Statistical Subareas. These restrictions are in place due to the rules of access and use of CCAMLR Data as agreed by it Member States. As a result, we are not permitted to publicly share the original raw data.

However, to ensure full reproducibility of our analyses, we have provided cleaned and anonymized data templates, along with all relevant R code, in the following publicly accessible GitHub repository: https://github.com/MauroMardones/LBSPR_Krill

This repository is also linked in the Supporting Information 1 under the "Code Repository" section. We believe this solution allows other researchers to fully replicate the analytical workflow and verify the results.

We thank you for your understanding regarding these data-sharing constraints.

Obs 5: Please upload a copy of Supporting Information Figure/Table/etc. Supporting Information 1 and 2 which you refer to in your text on page 36.

Reply:

Supporting Information 1 and 2 were originally provided during the initial submission as web links to the author's GitHub page, as they are part of the project’s reproducible workflow. Although these links remain publicly accessible and are cited accordingly in the Supporting Information section in manuscript (page 36), we have now also uploaded Supporting Information 1 and 2 in PDF format to the submission portal, as requested by the editor. This ensures the material is available both through the manuscript references and directly via the journal platform.

Obs 6: Please review your reference list to ensure that it is complete and correct. If you have cited papers that have been retracted, please include the rationale for doing so in the manuscript text, or remove these references and replace them with relevant current references. Any changes to the reference list should be mentioned in the rebuttal letter that accompanies your revised manuscript. If you need to cite a retracted article, indicate the article’s retracted status in the References list and also include a citation and full reference for the retraction notice.

[Note: HTML markup is below. Please do not edit.]

Reply:

We have reviewed and updated the formatting of these references without altering their position in the manuscript.

*25.	Hill S, Hinke J, Ratcliffe N, Trathan P, Watters G. WG-EMM-2019/28 Advances are urgently needed in providing regular estimates of krill stock status based on the available data. CCAMLR; 2019.*

*40.	Molles MC, Sher A. Ecology: Concepts and applications. 8th ed. New York, NY: McGraw-Hill Education; 2019.*

*56.	Mace PM. A new role for MSY in single-species and ecosystem approaches to fisheries stock assessment and management. Fish and Fisheries. 2001;2: 2–32. doi:*[*10.1046/j.1467-2979.2001.00033.x*](https://doi.org/10.1046/j.1467-2979.2001.00033.x)

*76.	Maschette D, Wotherspoon S, Kawaguchi S, Ziegler P. WG-FSA-2021/39 29. Grym assessment for Subarea 48.1 Euphausia superba populations. CCAMLR; 2021.*

**Reviewer 1 Observations and Replys**

Reviewer #1: This research is particularly interesting because it explores the use of LBSPR spatially. The manuscript is well written and presented. However, there are some areas that still need improvement to enhance the quality of this paper.

Obs 1: Adding an insert map to make it easier for the reader to know the location of the research and others that can be seen in the comments section in the reviewed manuscript file. - line 154 (Fig 1)

Reply: We have now added a new Fig 1.

Obs 2: Specify the length type used. Is it carapace/total/body length? - line 162

Reply: Detailed in lines 178-180;
 *“…International Scientific Observation (SISO). Krill length compositions (total length (TL) measured from the anterior edge of the eye to the posterior end of the telson, excluding terminal spines)…”*

Obs 3: Use the growth parameter symbol k (lower case) consistently to differentiate from carrying capacity (K) as well as natural mortality as M (capital) - line 230-231 and 253.

Reply: We have now c**orrected this throughout the manuscript, distinguishing between K (carrying capacity) and k (von Bertalanffy growth coefficient), and capitalizing natural mortality (M) accordingly yin all document.**

Obs 4: Please re-check the appropriate location as commented in line 324 and 327.

Reply: Thanks for the observation. The reviewer is correct—strata South West were properly added with the characteristics described in the corresponding paragraph.

5. Line 331 - which one did you uses as LRP 0.25 or 0.2?

Reply: Thanks for pointing this out. We can confirm that the value used as the Limit Reference Point (LRP) was 0.20. The manuscript has been corrected accordingly.

Any changes to the reference list should be mentioned in the rebuttal letter that accompanies your revised manuscript. If you need to cite a retracted article, indicate the article’s retracted status in the References list and also include a citation and full reference for the retraction notice.
