# Supplementary material for: Disparate estimates of intrinsic productivity for Antarctic krill across small spatial scales under a rapidly changing ocean
Source: PLoS One. 2026 Jan 30;21(1):e0322671. doi: 10.1371/journal.pone.0322671 (PMC12857959; doi:10.1371/journal.pone.0322671)
Supplement: Attachment — Submitted filename: Response to Reviewers_2a.docx [file pone.0322671.s005.docx]

**Response to Reviewers Cover Statement**

We thank the Academic Editor and both reviewers for their thorough evaluation of our manuscript and for the constructive recommendations provided. We carefully considered each comment and undertook an extensive revision of the manuscript to improve clarity, methodological transparency, and alignment with PLOS ONE’s standards. In particular, we substantially clarified the structure and functioning of the model, expanded the discussion of data limitations and potential biases, simplified the narrative to enhance readability, and revised ambiguous or non-standard English expressions. We also improved figure resolution and reduced reliance on acronyms, adding definitions where necessary.

All reviewer and editor comments have been addressed point by point in the sections below, and corresponding changes have been implemented in both the tracked-changes manuscript and the clean revised version. We sincerely appreciate the feedback, which has significantly strengthened the manuscript.

**Editor Observations and Responses**

**Comments to the Author**

1. If the authors have adequately addressed your comments raised in a previous round of review and you feel that this manuscript is now acceptable for publication, you may indicate that here to bypass the “Comments to the Author” section, enter your conflict of interest statement in the “Confidential to Editor” section, and submit your "Accept" recommendation.

Reviewer #1: All comments have been addressed

Reviewer #2: (No Response)

2. Is the manuscript technically sound, and do the data support the conclusions?

The manuscript must describe a technically sound piece of scientific research with data that supports the conclusions. Experiments must have been conducted rigorously, with appropriate controls, replication, and sample sizes. The conclusions must be drawn appropriately based on the data presented.

Reviewer #1: Yes

Reviewer #2: Partly

Reply: We appreciate the reviewer’s comment. The methodological details, including data templates, code, and all procedures used to ensure analytical rigor, are fully clarified in Supporting Information 1, where we provide complete documentation of the workflow and datasets employed.

3. Has the statistical analysis been performed appropriately and rigorously?

Reviewer #1: Yes

Reviewer #2: I Don't Know

Reply: We thank the reviewer for raising this point. The statistical formulas underlying the model are fully presented in Supporting Information 1, and all code used to implement the analyses is embedded within the manuscript to ensure full transparency and reproducibility.

4. Have the authors made all data underlying the findings in their manuscript fully available?

The [PLOS Data policy](http://track.editorialmanager.com/CL0/http:%2F%2Fwww.plosone.org%2Fstatic%2Fpolicies.action%23sharing/1/010f019a39292797-339e5223-fbc8-40a5-892f-11a1c4666dc3-000000/E_L1gBQrGt-5zOtBvBPeI9qFIVga3xeKuIWxA7nL2Tc=235) requires authors to make all data underlying the findings described in their manuscript fully available without restriction, with rare exception (please refer to the Data Availability Statement in the manuscript PDF file). The data should be provided as part of the manuscript or its supporting information, or deposited to a public repository. For example, in addition to summary statistics, the data points behind means, medians and variance measures should be available. If there are restrictions on publicly sharing data—e.g. participant privacy or use of data from a third party—those must be specified.

Reviewer #1: Yes

Reviewer #2: No

Reply: Thank you for your comment. As noted in the Data Availability Statement, he original raw data underlying this study are managed by the Commission for the Conservation of Antarctic Marine Living Resources (CCAMLR) and are subject to legal and institutional restrictions under the CCAMLR Rules for Access and Use of Data. As a result, the authors do not have permission to publicly share the original raw datasets.

However, the minimal processed and anonymized datasets required to replicate the analyses and results presented in this study are publicly available in a stable repository. These processed data, together with the complete analytical workflow, have been deposited in Zenodo and can be accessed via the following DOI: <https://doi.org/10.5281/zenodo.17936869>. The processed datasets archived in Zenodo consist of aggregated and atomized length–frequency data derived from the original CCAMLR datasets exclusively for the purpose of reproducibility and do not substitute the original raw data holdings.

Also, and to fully comply with PLOS data requirements while respecting these restrictions, we have made all processed data and the complete code needed to replicate the analysis are provided in the **Code Repository** section of **Supporting Information 1**, which offers a step-by-step guide for reproducing the results. In response to the reviewer’s observation, we have updated the README file in the code repository (<https://github.com/MauroMardones/LBSPR_Krill> linked to the Zenodo archive) to clearly explain the structure of the repository, the purpose of each script and dataset, and the workflow required to run the analysis.

5. Is the manuscript presented in an intelligible fashion and written in standard English?

PLOS ONE does not copyedit accepted manuscripts, so the language in submitted articles must be clear, correct, and unambiguous. Any typographical or grammatical errors should be corrected at revision, so please note any specific errors here.

Reviewer #1: Yes

Reviewer #2: No

Reply: The manuscript has undergone a thorough language revision to ensure clarity and fluency. All sections were revised to ensure that the text is clear, concise, and written in standard academic English, without introducing any structural changes to the original content. Although the reviewers did not specify which particular passages required language improvement, we have carefully revised the entire manuscript to address any potential issues. All modifications have been incorporated in the revised version using Track Changes to facilitate the review of the language improvements and ensure full transparency in the revision process.

6. Review Comments to the Author

Please use the space provided to explain your answers to the questions above. You may also include additional comments for the author, including concerns about dual publication, research ethics, or publication ethics. (Please upload your review as an attachment if it exceeds 20,000 characters)

Reviewer #1: All my comments have been addressed well. From my side, this manuscript was ready for publication.

Reviewer #2: This manuscript presents results of applying a length-based model to estimate secondary production by Antarctic krill. The authors find that the model produces different estimates of production in different regions, and different years, and also produces different results when parameters are varied within plausible bounds. The authors discuss relevance to fisheries management.

**Reviewer 2 Observations**

**Obs1**: It was unclear to me how the model worked, which made it difficult to evaluate the results.

Reply: We appreciate the reviewer’s observation and understand that the model description may not have been sufficiently clear in the original version. To improve the transparency and understanding of how the LBSPR model works and how was implemented for Antarctic krill, we have revised the Methods section and expanded the supplementary material.

In the revised version, we have added a set of equations in **Supporting Information 1** that describe the full computational workflow of the SPR estimation process, from model inputs to intermediate calculations and final outputs. These equations outline the structure of the model and the sequence of operations in R codes used to obtain the estimates presented in the manuscript.

**Obs 2**: I was also concerned by the assertion that length is a proxy for growth.

Reply: We thank the reviewer for this comment. In fisheries science, using *length* as a proxy for *growth* is not only standard practice but foundational to the most widely applied growth and life-history models. Generally, growth can be defined as the change in length or weight over the life of an individual, and it directly influences survival, sexual maturity, reproductive success, movement, and migration (Peters, 1986). In this sense, the most commonly used model to describe somatic growth through time, **von Bertalanffy Growth Function (VBGF)**, is explicitly length-based. This framework relies on observed lengths to estimate growth trajectories and key biological parameters such as the growth coefficient (*k*) and the asymptotic length (*L∞*). Thus, length is intrinsically embedded in the way growth is modeled, interpreted, and applied in fishery science. To strengthen this point, we have expanded the manuscript text (line 231) supporting the use of length as a proxy for growth in population assessments, including those related to the VBGF and length-based methods:

- Beverton, R. J. H., & Holt, S. J. (1957). *On the Dynamics of Exploited Fish Populations.* Fishery Investigations Series II, Vol. 19.
- Pauly, D. (1980). On the interrelationships between natural mortality, growth parameters, and environmental temperature in 175 fish stocks. *ICES Journal of Marine Science*, 39(2), 175–192.
- Sparre, P., & Venema, S. C. (1998). *Introduction to Tropical Fish Stock Assessment. Part 1: Manual.* FAO Fisheries Technical Paper 306/1.
- Peters, R. H. (1986). *The Ecological Implications of Body Size*. Cambridge University Press. (This reference have been added to the revised manuscript)
- Kell LT, Minto C, Gerritsen HD. Evaluation of the skill of length-based indicators to identify stock status and trends. ICES Journal of Marine Science. 2022;79: 1202–1216. doi:10.1093/icesjms/fsac043

**Obs 3:** It is great to see fisheries data used, but I felt the manuscript was missing some mention of the limitations and potential biases in this data.

Reply: We thank the reviewer for this helpful comment. In response, we have added a dedicated section in the Discussion called **Limitations and caveats of length-based assessment methods** addressing the limitations and potential uncertanty and biases associated with the fisheries data used in this study. This new text outlines key sources of uncertainty such as data scarse, representativeness of length data, and life history parameters. We have also included a discussion of the limitations of the LBSPR model itself, particularly regarding how estimation performance can vary depending on the stock’s status. Specifically, we now describe how SPR estimates may become biased when the population is either heavily depleted or lightly exploited, and how these conditions can influence the reliability of the derived biological indicators. We believe these additions aim to provide a more balanced interpretation of the results and to clarify the contexts in which the model performs robustly and where caution is needed.

**Obs 4:** The title doesn't appear to reflect the contents, as most of the regions studied showed no evidence of temporal trends which could be associated with environmental change.

Reply: Thank you for this comment. We agree that the observed temporal trends were not consistent across all regions, and in several cases no clear patterns emerged that could be directly linked to environmental change. However, the objective of the manuscript is not to demonstrate uniform temporal trends, but rather to highlight the high spatial variability in intrinsic productivity estimates and to discuss how such variability can occur even under a rapidly changing ocean.

The title, “Disparate estimates of intrinsic productivity for Antarctic krill across small spatial scales under a rapidly changing ocean,” emphasizes this central message, that intrinsic productivity varies markedly across neighbouring regions, and that this heterogeneity is highly relevant in the context of environmental change, even if not all regions show directional temporal trends. To improve clarity, we have adjusted the Introduction and Discussion to explicitly state that the primary focus of the study is spatial contrasts in intrinsic productivity rather than uniform temporal patterns.

**Obs 5:** I found the manuscript difficult to read. The authors have clearly put a lot of effort into using elegant scientific language, but I found the unnecessary verbiage obscured the main messages of the manuscript. The manuscript could be much improved by simplifying the language and focusing on clearly communicating the approach and results.

Reply: Thank you for this comment. We appreciate the concern regarding readability. In response, we conducted a detailed review of the entire manuscript, focusing on simplifying the language, reducing overly elaborate phrasing, and improving the clarity and flow of the text without compromising scientific accuracy. Because the reviewer did not specify which sections were problematic, we carried out a comprehensive, line-by-line assessment to identify and refine any wording that could obscure the main messages.

We also acknowledge that modelling-focused manuscripts can naturally be dense and demanding to read due to the technical nature of the methods and results. Even so, we made a concerted effort to improve clarity wherever possible, and we believe the manuscript is now more fluid and easier to follow. These improvements are highlighted in the tracked-changes file and also incorporated into the final revised manuscript.

**Obs 6:** In places, the language is also not exactly standard English (e.g. "Along the Southern Ocean" [the southern ocean is roughly a circle, one doesn't typically refer to going along circles], "Krill is the largest marine biomass on Earth" [krill may have or represent the largest biomass, but one doesn't typically use "is" in this context]).

Reply: Thank you for the observation. The expressions noted have been corrected in the revised manuscript, along with similar phrasing issues identified during our review (Lines 48 and 58)

**Obs 7:** The manuscript also contains many acronyms - readability could be improved by using these more judiciously, or if they are necessary, perhaps including a table or glossary.

Reply: We appreciate the reviewer’s observation. In response, we carefully reviewed the use of acronyms throughout the manuscript to ensure they are employed only where necessary. Additionally, Supporting Information 1 now includes a **glossary of technical terms** that defines the main parameters, variables, fishery concepts, and acronyms used in the analysis. We believe these improvements enhance the clarity of the document and strengthen the transparency and interpretability of the results.

**Obs 8:** The figures were also rather blurry in the proofs; perhaps this is an issue specific to the manuscript proofs, but it might be worth double checking, and considering if the plots could be output in a file format which better preserves resolution (e.g. SVG).

Reply: Thank you for your observation. The figures were included in the format required by the journal (.tiff). When downloaded directly from the PDF submmitted, they can be viewed at a higher resolution, which should address the issue you observed in the proofs.

7. PLOS authors have the option to publish the peer review history of their article ([what does this mean?](https://track.editorialmanager.com/CL0/https:%2F%2Fjournals.plos.org%2Fplosone%2Fs%2Feditorial-and-peer-review-process%23loc-peer-review-history/1/010f019a39292797-339e5223-fbc8-40a5-892f-11a1c4666dc3-000000/4WQNGMmiovIZ7v-7On-cY0KTrfTailnBh-jmYiGJGjw=235)). If published, this will include your full peer review and any attached files.


If you choose “no”, your identity will remain anonymous but your review may still be made public.


**Do you want your identity to be public for this peer review?** For information about this choice, including consent withdrawal, please see our [Privacy Policy](https://track.editorialmanager.com/CL0/https:%2F%2Fwww.plos.org%2Fprivacy-policy/1/010f019a39292797-339e5223-fbc8-40a5-892f-11a1c4666dc3-000000/YYRsdzQn-JGNOPiHTLsZV5nM6j5uQbEGHJh2kvPnWak=235).

Reviewer #1: No

Reviewer #2: No

[NOTE: If reviewer comments were submitted as an attachment file, they will be attached to this email and accessible via the submission site. Please log into your account, locate the manuscript record, and check for the action link "View Attachments". If this link does not appear, there are no attachment files.]

To ensure your figures meet our technical requirements, please review our figure guidelines: <https://journals.plos.org/plosone/s/figures>

You may also use PLOS’s free figure tool, NAAS, to help you prepare publication quality figures: <https://journals.plos.org/plosone/s/figures#loc-tools-for-figure-preparation>.

NAAS will assess whether your figures meet our technical requirements by comparing each figure against our figure specifications.
